# 1,2-Dimethylhydrazine: MAK Value Documentation, addendum – Translation of the German version from 2021

**DOI:** 10.34865/mb54073e9_4ad

**Published:** 2024-12-23

**Authors:** Andrea Hartwig, MAK Commission

**Affiliations:** 1 Institute of Applied Biosciences. Department of Food Chemistry and Toxicology. Karlsruhe Institute of Technology (KIT) Adenauerring 20a, Building 50.41 76131 Karlsruhe Germany; 2 Permanent Senate Commission for the Investigation of Health Hazards of Chemical Compounds in the Work Area. Deutsche Forschungsgemeinschaft, Kennedyallee 40, 53175 Bonn, Germany. Further information: Permanent Senate Commission for the Investigation of Health Hazards of Chemical Compounds in the Work Area | DFG

**Keywords:** 1,2-dimethylhydrazine, carcinogenicity, colon carcinoma, germ cell mutagenicity, toxicity, DNA methylation

## Abstract

The German Commission for the Investigation of Health Hazards of Chemical Compounds in the Work Area (MAK Commission) has re-evaluated the occupational exposure limit value (maximum concentration at the workplace, MAK value) of 1,2-dimethylhydrazine [540-73-8] considering all toxicological end points. Relevant studies were identified from a literature search. Chronic and subchronic exposure induced adverse effects on the liver, heart and kidneys of mice and the liver and bile ducts of mini-pigs and guinea pigs. In dogs, 1,2-dimethylhydrazine caused adverse effects on the liver. The critical effect of 1,2-dimethylhydrazine is its carcinogenic potential. In carcinogenicity studies, 1,2-dimethylhydrazine induced various intestinal and vascular tumours such as haemangiosarcomas in addition to lung tumours in rodents after oral and intraperitoneal application. Particularly noteworthy is the high incidence of colon carcinomas in rats, which was observed both after acute and chronic application. Additionally, tumours of the digestive system were caused in hamsters and monkeys after subcutaneous or intramuscular injection. On the basis of the carcinogenic effects induced in several animal species, 1,2-dimethylhydrazine remains classified in Carcinogen Category 2. It is not possible to derive a MAK value. Enzymatic activation of 1,2-dimethylhydrazine leads to highly reactive metabolites, such as the well-known procarcinogen methylazoxymethanol, which are able to methylate DNA. The substance is clastogenic and mutagenic in somatic cells in vitro and in vivo. Additionally, in mouse testes, 1,2-dimethylhydrazine was found to inhibit DNA synthesis after oral application and to methylate DNA after subcutaneous application. Therefore, the substance has been classified in Germ Cell Mutagenicity Category 3 A. Although no valid studies considering the dermal absorption of 1,2-dimethylhydrazine are available, the “H” designation has been retained because of the low dermal LD_50_ values in animals and the potential for genotoxic effects after dermal application. Although no studies considering the sensitizing potential of 1,2-dimethylhydrazine are available, the “Sh” designation has been retained because of its structural similarity with the known contact allergen hydrazine.

**Table:** 

**MAK value**	**–**
**Peak limitation**	**–**
	
**Absorption through the skin (1979)**	**H**
**Sensitization (1979)**	**Sh**
**Carcinogenicity (1980)**	**Category 2**
**Prenatal toxicity**	**–**
**Germ cell mutagenicity (2020)**	**Category 3 A**
	
**BAT value**	**–**
	
Synonyms	*N*,*N′*-dimethylhydrazine symmetrical dimethylhydrazine
Chemical name (IUPAC)	1,2-dimethylhydrazine
CAS number	540-73-8
Structural formula	H_3_C–NH–NH–CH_3_
Molecular formula	C_2_H_8_N_2_
Molar mass	60.10 g/mol
Melting point	–9 °C (IFA 2019)
Boiling point at 1004 hPa	81 °C (IFA 2019)
Density at 20 °C	0.83 g/cm^3^ (IFA 2019)
Vapour pressure at 20 °C	70.8 hPa (NCBI 2020)
log K_OW_	–0.54 (calculated; NCBI 2020)
Solubility	miscible with water (NCBI 2020)
**1 ml/m^3^ (ppm) ≙ 2.50 mg/m^3^**	**1 mg/m^3^ ≙ 0.401 ml/m^3^ (ppm)**
	
Hydrolytic stability	no data
Production	from dibenzoylhydrazine or via electrosynthesis from nitromethane (ATSDR 1997)
Uses	research chemical (IARC 1999)

Documentation for 1,2-dimethylhydrazine was published in 1973 (Henschler 1973, available in German only). In this addendum, the substance and in particular its potential carcinogenicity are re-evaluated on the basis of the more extensive set of data that has become available from studies carried out since 1972.

## 1 Toxic Effects and Mode of Action

1,2-Dimethylhydrazine is not irritating to the skin of guinea pigs and rabbits. However, it is irritating to the eyes of rabbits.

1,2-Dimethylhydrazine caused mainly colon tumours in rats, mice, hamsters and monkeys by various routes of administration. Even a single gavage dose of 30 mg 1,2-dimethylhydrazine dihydrochloride/kg body weight (equivalent to 13.6 mg 1,2-dimethylhydrazine/kg body weight) induced colon tumours in rats. Furthermore, blood vessel tumours, tumours of the perianal glands, and uterine and lung tumours formed in mice after oral, intraperitoneal and subcutaneous administration. After chronic and subchronic exposure, the target organs in various species were the intestine, liver, heart, kidneys, adrenal glands and bile duct.

Valid studies of developmental toxicity are not available for 1,2-dimethylhydrazine.

Data for sensitization are not available.

1,2-Dimethylhydrazine has mutagenic and DNA-damaging effects (including DNA adduct formation) in bacteria and mammalian cells in vitro. Studies in vivo confirm adduct formation, also in the testes, and DNA-damaging effects in mammalian cells. Although none of the available tests allows a differentiation to be made between aneugenic and clastogenic effects, a clastogenic effect is to be assumed in the light of all the available data. In addition, there is evidence of mutagenicity in mammalian somatic cells in vivo.

## 2 Mechanism of Action

### 2.1 Neurotoxicity

In acute studies, convulsions have been reported in animals at near-lethal doses (Kennedy 2012; Rothberg and Cope 1956). However, the mechanism of CNS toxicity postulated for monomethylhydrazine and 1,1-dimethylhydrazine via hydrazone formation with derivatives of vitamin B6 is not possible for 1,2-dimethylhydrazine on account of its structure (ATSDR 1997). It is unclear whether the reported convulsions result from specific CNS toxicity.

### 2.2 Genotoxic effects and carcinogenicity

Genotoxic and carcinogenic effects of 1,2-dimethylhydrazine can be attributed to highly reactive intermediates formed during metabolism. In addition to methyl radicals, the methyldiazonium ion should be mentioned here, which is formed from one of the main intermediates, methyl azoxymethanol. The methyldiazonium ion can methylate DNA. Methylation of DNA bases has been demonstrated in in vivo and in vitro studies (Section 5.6). O6-Methylguanine and O4-methylthymidine can lead to mismatches of thymine and guanine in DNA and, moreover, cause mutations (G:C → A:T), as has been demonstrated with 1,2-dimethylhydrazine (Horsfall et al. 1990). Mutations are closely associated with carcinogenic effects of the substance (IARC 1999; Perše and Cerar 2005). Mutations in colon cells and the formation of colon tumours in the distal region in rodents, particularly in rats, occur after only a single dose of 1,2-dimethylhydrazine (ATSDR 1997; IARC 1999; Newell and Heddle 2004). The carcinogenicity of 1,2-dimethyl­hydrazine is based on the development of dysplasia of aberrant crypts in the colon, which develop into adenomas and adenocarcinomas (Jikihara et al. 2015).

### 2.3 Local irritation

Due to its alkaline properties in aqueous solution, 1,2-dimethylhydrazine is regarded as corrosive and irritating to the skin, eyes and respiratory tract (Kennedy 2012; NIOSH 1978). However, according to experimental data, the irritant effect is not very pronounced (Section 5.3).

## 3 Toxicokinetics and Metabolism

### 3.1 Absorption, distribution, elimination

Data from human and animal studies after ingestion or dermal application are not available.

After subcutaneous injection of 21 mg ^14^C-1,2-dimethylhydrazine/kg body weight in F344 rats, 14% was exhaled as azomethane and 11% as carbon dioxide within 24 hours. At 200 mg/kg body weight, the corresponding proportions were 23% and 4% within the same time period. Most of the azomethane was exhaled in the first 6 hours (Fiala et al. 1976). Furthermore, trace amounts of azoxymethane and methylazoxymethanol were detected in the urine of exposed animals (Fiala 1977).

Male SD rats were given subcutaneous injections of ^14^C-1,2-dimethylhydrazine of 20 mg/kg body weight. After 12 hours, about 16% had been exhaled as azomethane and about 22% as carbon dioxide, most within 6 hours (Harbach and Swenberg 1981).

Albino rats given subcutaneous injections of 21 mg ^3^H-1,2-dimethylhydrazine/kg body weight exhaled about 24% as carbon dioxide within 24 hours and 10% was excreted in the urine. Radioactivity was detected in the blood, bile, urine, and all parts of the digestive tract as early as 15 to 30 minutes after injection. This shows that the substance and possible degradation products are rapidly distributed in the body of the rat (Pozharisski et al. 1976, 1977).

After a subcutaneous injection of 200 mg/kg body weight, only 0.9% of the administered radioactivity was found in the bile of rats. Thus, the faeces were excluded as the main route of excretion (Hawks and Magee 1974).

The following half-lives were estimated for the kinetics of the conversion of ^14^C-1,2-dimethylhydrazine and its metabolites in perfused rat liver: 1,2-dimethylhydrazine → azomethane: 21.8 minutes; azomethane → azoxymethane: 1.5 minutes; azoxymethane → methylazoxymethanol: 41.5 minutes. Methylazoxymethanol was relatively stable with a half-life of 611 minutes. There was little biliary excretion, demonstrating that the transformation to glucuronides was of secondary importance (Wolter and Frank 1982).

Using the perfusion technique, it was found in vivo that the substance was rapidly absorbed by the colon of the Sprague Dawley (SD) rat. The rate of absorption was linear to the concentration used and absorption was increased by bile acids and hydroxy fatty acids (Meshkinpour et al. 1985).

### 3.2 Metabolism

In in vivo studies, the following steps in the metabolic degradation of 1,2-dimethylhydrazine have been demonstrated: after dehydrogenation of the substance to azomethane, *N*-oxidation to azoxymethane and *C*-oxidation to methylazoxy­methanol, which decomposes to the methyldiazonium ion, occur. Furthermore, carbon dioxide is formed (ATSDR 1997). In SD rats, after intraperitoneal doses of 20, 60, or 180 mg 1,2-dimethylhydrazine/kg body weight, also exhaled ethane was detected as a degradation product of the substance. The amount exhaled was dose-dependent. The formation of ethane was attributed to the dimerization of methyl radicals formed in the metabolism of 1,2-dimethylhydrazine (Kang et al. 1988).

In vitro studies demonstrated the formation of formaldehyde, nitrogen and methanol from methylazoxymethanol in aqueous solution. Methyldiazonium hydroxide was postulated as the methylating intermediate (Nagasawa et al. 1972). With alcohol dehydrogenase or cytochrome P450 (CYP), methylazoxymethanol is converted into methylazoxyformaldehyde, which is then converted into methyldiazonium hydroxide (Feinberg and Zedeck 1980; Sohn et al. 1991).

In rat liver perfusate, azomethane, azoxymethane, and methylazoxymethanol were detected as degradation products of 1,2-dimethylhydrazine (Wolter and Frank 1982). Also in colon epithelial cells of rats, metabolism of 1,2-dimethylhydrazine to azoxymethane, methylazoxymethanol, and a reactive species was shown (Glauert and Bennink 1983). Similarly, the formation of formaldehyde has been demonstrated with human microsomes from colon cells and ­human colon tumour cells, and inhibition of metabolism by CYP inhibitors has been seen (Newaz et al. 1983). Using rat microsomes from liver and lung cells, the formation of azomethane and formaldehyde was determined after the addition of 1,2-dimethylhydrazine. Oxidation of the nitrogen-to-nitrogen bond of 1,2-substituted hydrazines catalysed by microsomal CYP enzymes and by mitochondrial monoamine oxidases was shown to lead to the formation of azo compounds (Erikson and Prough 1986). Methyl radicals were formed from 1,2-dimethylhydrazine using rat liver micro­somes and rat hepatocytes (Albano et al. 1993; Tomasi et al. 1987). The generation of methyl radicals has also been established by the activation of 1,2-dimethylhydrazine by horseradish peroxidase (Augusto et al. 1985; Netto et al. 1988).

The formation of azomethane, azoxymethane and methylazoxymethanol takes place as an enzymatically mediated reaction primarily in the liver, but also in other metabolically active tissues. The methylazoxymethanol formed can be conjugated also with glucuronic acid and transported to the bile and thus to the intestine. There, the conjugate can be hydrolysed so that free methylazoxymethanol is once more present (Fiala 1977). However, this can be transported via the bloodstream directly into the epithelial cells of the intestine. These cells can also independently convert 1,2-dimethylhydrazine into carcinogenic metabolites (Perše and Cerar 2005).

The reactive intermediates (methyl radicals and methyldiazonium ions) formed in the metabolism of 1,2-dimethylhydrazine cause the methylation of DNA bases. The methyldiazonium ion probably methylates by an S_N_2 mechanism. DNA methylation has been detected in in vivo (Table 3) and in vitro experiments (Autrup et al. 1980 a; Harris et al. 1977; Kumari et al. 1985; Netto et al. 1988). The DNA adducts were N7-methylguanine and O6-methylguanine and, to a lesser extent, N3-methyladenine and O4-methylthymidine. C8-Methylguanine was detected as a result of radical methylation (Netto et al. 1992).

Selenium decreased DNA base methylation in the liver in rats but increased it in the colon. The administration of selenium increased also the levels of exhaled azomethane and decreased the levels of exhaled carbon dioxide. The data indicate that selenium inhibits hepatic metabolism while increasing extrahepatic metabolism. Although DNA base methylation was increased in colon cells, treatment with selenium decreased the incidence of colon tumours. This was attributed to decreased DNA synthesis by selenium as a marker of cell proliferation in colon cells (Harbach and Swenberg 1981). 

Metabolic activation of the substance in human and rodent tissues is similar and results in the same DNA adducts: O6-methylguanine and N7-methylguanine were detected at the same ratio in rat and human intestinal cells. The frequency of DNA adducts occurred in the following order: N7-methylguanine > O6-methylguanine > N3-methyladenine (Autrup et al. 1980 b).

**Fig.1:**
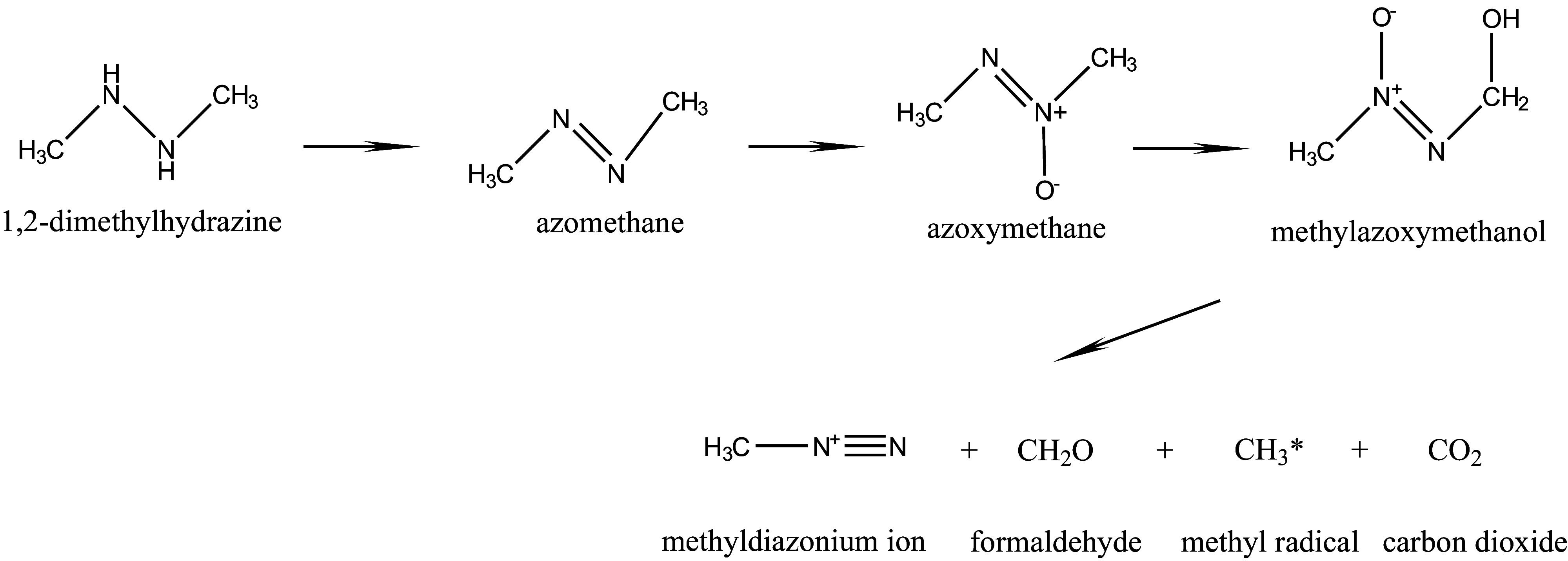
Metabolism of 1,2-dimethylhydrazine (according to Fiala 1977)

The metabolism of 1,2-dimethylhydrazine is shown in Figure 1.

### 3.3 Summary

1,2-Dimethylhydrazine is distributed throughout the mammalian body and is metabolized primarily, but not exclusively, in the liver. The substance is oxidized by CYP and monoamine oxidases to azomethane, then to azoxymethane and finally to methylazoxymethanol; this, in turn, is converted into methyldiazonium ions, formaldehyde and carbon dioxide. In addition, also methyl radicals are formed, which can dimerize to ethane. As the methyldiazonium ion has a strong alkylating effect, it forms DNA adducts at different positions of DNA bases as well as adducts with proteins and RNA. 1,2-Dimethylhydrazine is oxidized also by human cells to reactive species. Similar DNA adducts are formed in human and animal cells. The main degradation products of 1,2-dimethylhydrazine (carbon dioxide and azomethane) are primarily exhaled and other transformation products are excreted with the urine, whereas the faeces are not a major route of excretion.

## 4 Effects in Humans

It seems likely that 1,2-dimethylhydrazine has skin sensitization potential, since hydrazine is a strong contact allergen and cross-reactions between hydrazine derivatives are known (Greim 1999). However, specific studies for 1,2-dimethylhydrazine are not available.

There are no data available for other end points.

## 5 Animal Experiments and in vitro Studies

### 5.1 Acute toxicity

#### 5.1.1 Inhalation

For rats, the 4-hour LC_50_ is between 280 and 400 ml/m^3^ (ATSDR 1997).

#### 5.1.2 Oral administration

For male and female mice, the LD_50_ values after oral administration are 11.7 and 27.1 mg/kg body weight, respectively (Visek et al. 1991).

#### 5.1.3 Dermal application

After application of the substance to the shaved dorsal skin of rabbits and guinea pigs, the LD_50_ values were 563 and 158 µl/kg body weight (467 and 131 mg/kg body weight), respectively. Tremor and convulsions occurred at near-lethal doses (Rothberg and Cope 1956).

#### 5.1.4 Intravenous, intraperitoneal and subcutaneous injection

The LD_50_ values after intravenous injection were 100 mg/kg body weight for dogs (no other details) and 176 mg/kg body weight for rats (no other details), and after intraperitoneal administration, 35 mg/kg body weight for mice (no other details) and 163 mg/kg body weight for rats (no other details). After subcutaneous injection, the LD_50_ values were 220 mg/kg body weight for rats (no other details) and 24 mg/kg body weight for mice (no other details, NCBI 2020).

The acute effect of the substance on intestinal mucosal cells was studied after a single intrarectal injection of 25 mg/kg body weight in SD rats. Inhibition of DNA synthesis was observed (12 and 24 hours after injection). Five days after injection, a reduction in the amount of adenosine 3,5-monophosphate and increased protein kinase activity in the cytosol and thus a possible effect on cell proliferation were observed (DeRubertis and Craven 1980).

Groups of 3 male and 3 female NMRI and ASI mice were given a subcutaneous injection of 15 mg 1,2-dimethylhydrazine/kg body weight, and 12 Wistar rats of 200 mg/kg body weight. Forty-eight hours after exposure, centrilobular necrosis of the liver was observed in both mice and rats, though it was more pronounced in rats. Six hours after treatment, morphological changes in small intestine and colon cells were observed in rats (irregular nuclear alignment, nuclear fragmentation) and to a lesser extent in mice. These effects were most pronounced 24 hours after exposure. In the rat liver, nucleolar microsegregation, disaggregation of ribosomes, inhibited incorporation of ^3^H-leucine into liver proteins, the swelling of mitochondria and changes in their shape were observed 6 hours after exposure. The structural changes at the subcellular level were very similar to those caused by dimethylnitrosamine (Hawks et al. 1974).

#### 5.1.5 Summary

The acute toxicity of 1,2-dimethylhydrazine is moderate to severe. Of the species studied, mice were the most sensitive. Intestinal and liver cells of rats and mice developed altered subcellular structures, and liver protein biosynthesis was inhibited. Liver cell necrosis occurred in mice and rats.

### 5.2 Subacute, subchronic and chronic toxicity

#### 5.2.1 Inhalation

There are no data available.

#### 5.2.2 Oral administration

To investigate any species differences in the toxicity and carcinogenicity of 1,2-dimethylhydrazine, minipigs, dogs, guinea pigs and rats were given 1,2-dimethylhydrazine dihydrochloride by gavage. The doses were converted to equivalent doses of 1,2-dimethylhydrazine. Minipigs and guinea pigs were given the substance in 10 weekly doses of 13.6 or 27.1 mg 1,2-dimethylhydrazine/kg body weight (1.94 or 3.87 mg/kg body weight and day). Dogs were given 2.26, 6.8, 13.6 or 27.1 mg 1,2-dimethylhydrazine/kg body weight in up to 10 weekly doses (0.32, 0.97, 1.94, 3.87 mg/kg body weight and day). Ten rats per group received 13.6 mg/kg body weight in 4 or 8 weekly doses (1.94 mg/kg body weight and day). The animals were observed for up to 18 months after exposure. One minipig in the low dose group and 5 in the high dose group died. Animals in both dose groups exhibited haemorrhage (especially in the intestine) and haemorrhagic degeneration, necrosis, icterus, bile duct proliferation and hepatocellular megalocytosis in the liver. Focal megalocytosis and postnecrotic fibrosis were observed in the liver of the surviving minipigs after 18 months. All dogs died after doses of 1.94 mg/kg body weight and day and above, and hepatic degeneration and haemorrhage and karyolysis in hepatocytes were recorded. The dogs in the lowest dose group survived, but suffered ascites, icterus, postnecrotic hepatic fibrosis and haemosiderosis. Of the guinea pigs in the high dose group, only one survived with a bile duct carcinoma. The animals that died had developed bile duct hyperplasia and hepatic necrosis. Animals in the low dose group developed hepatic fibrosis, ascites, hepatomas and bile duct carcinomas. All rats had colon tumours, and 3 rats had carcinomas of the ear canal (Wilson 1976).

The dependence of the effects of 1,2-dimethylhydrazine dihydrochloride (converted to 1,2-dimethylhydrazine below) on the dietary protein concentration was investigated in feeding studies lasting 6 weeks or 5 months. The experiments were performed in 5 to 8-week-old B6C3F1 mice (C57BL/6N × C2H/HEN, F1). The animals were fed 0, 5.1, 10.2, 20.3, 40.7 or 81.3 mg 1,2-dimethylhydrazine/kg diet containing 10% or 40% protein for 6 weeks. Due to high mortality, the findings in the animals of the high dose group were not included. Based on the reported food intake per animal and the reported final body weights, the doses in the 10% protein diet group for female mice were 0, 0.72, 1.55, 2.63 and 4.47, and for male mice 0, 0.68, 1.4, 2.77 and 5.05 mg 1,2-dimethylhydrazine/kg body weight and day. The corresponding doses in the 40% protein group for female mice were 0, 0.71, 1.43, 2.71 and 4.31, and for male mice 0, 0.63, 1.33, 2.68 and 4.66 mg 1,2-dimethylhydrazine/kg body weight and day. Each group consisted of 10 female and 10 male mice. Body weights and body weight gains were reduced compared with the values for the control animals except in the lowest dose group. The relative liver, kidney and heart weights were decreased in a dose-dependent manner in both sexes at and above 10.2 mg 1,2-dimethylhydrazine/kg diet (about 1.5 mg/kg body weight and day). The NOAEL (no observed adverse effect level) was approximately 0.7 mg/kg body weight and day. In a 5-month study, 25 male B6C3F1 mice per group were treated with 0, 6.8, 13.6 or 20.3 mg 1,2-dimethylhydrazine/kg diet containing 10% or 40% protein. The daily intakes of the animals in the 10% protein diet group were 0, 0.026, 0.046, and 0.062 mg 1,2-dimethylhydrazine, respectively. Based on the reported food intake per animal and the final body weights of the mice, the doses were 0, 0.74, 1.64 and 2.69 mg 1,2-dimethylhydrazine/kg body weight and day. For animals in the 40% protein diet group, the doses were 0, 0.77, 1.69 and 2.68 mg 1,2-dimethylhydrazine/kg body weight and day, respectively. Again, dose-dependent reductions in food intake, body weights and organ weights were observed. The liver was the most sensitive target organ: decreased relative liver weights, focal centrilobular hepatocellular necrosis and haemosiderosis occurred even in the low dose group. At the higher doses, lobular disorganization and hepatocellular hypertrophy, bile duct hyperplasia, hepatic fibrosis, interstitial nephritis, pyelonephritis, adrenal hypertrophy and focal myocytolysis of the heart were diagnosed. In animals fed the high protein diet, the adverse effects were less pronounced. A NOAEL was not obtained (Visek et al. 1991). A summary of the results is given in Table 1.

**Tab.1:** Effects of 1,2-dimethylhydrazine (given as dihydrochloride) after repeated oral intake

Species, strain, number per group	Exposure	Findings	References
mouse, B6C3F1, 10 ♀, 10 ♂	**6 weeks** 0, 5.1, 10.2, 20.3, 40.7 mg 1,2-dimethylhydrazine/kg diet with 10% or 40% protein content; corresponding to: ♀: 0, 0.72, 1.55, 2.63, 4.47; ♂: 0, 0.68, 1.4, 2.77, 5.05 mg 1,2-dimethylhydrazine/kg body weight and day (diet with 10% protein content) ♀: 0, 0.71, 1.43, 2.71, 4.31; ♂: 0, 0.63, 1.33, 2.68, 4.66 mg 1,2-dimethylhydrazine/kg body weight and day (diet with 40% protein content)	**about 1.5 mg/kg body weight and above:** body weights ↓, body weight gains ↓, relative weights of liver, kidneys, heart ↓	Visek et al. 1991
rat, SD, 10 ♂, 6 controls	**4 or 8 weeks** 0, 13.6 mg 1,2-dimethylhydrazine/kg body weight, 1× weekly; corresponding to: 0, 1.94 mg/kg body weight and day, gavage, 9 months recovery period	**1.94 mg/kg body weight:** 4 and 8 weeks: colon tumours (16/20), 8 weeks: carcinoma of the ear canal (3/10)	Wilson 1976
mouse, B6C3F1, 25 ♂	**5 months** 0, 6.8, 13.6, 20.3 mg 1,2-dimethylhydrazine/kg diet with 10% or 40% protein content; corresponding to: 0, 0.74, 1.64, 2.69 mg 1,2-dimethylhydrazine/kg body weight and day (diet with 10% protein content), 0, 0.77, 1.69, 2.68 mg 1,2-dimethylhydrazine/kg body weight and day (diet with 40% protein content)	**about 0.7 mg/kg body weight and above:** body weights ↓, body weight gains ↓, liver: relative weights ↓, focal centrilobular necrosis, haemosiderosis; **1.6 mg/kg body weight and above:** relative kidney weights ↓, hepatic fibrosis, lobular disorganization, liver cell hypertrophy, bile duct hyperplasia, interstitial nephritis, pyelonephritis, adrenal gland hypertrophy, focal myocytolysis	Visek et al. 1991
guinea pig, Hartley, 6 ♂	**up to 10 weeks** 0, 13.6, 27.1 mg 1,2-dimethylhydrazine/kg body weight, 1× weekly; corresponding to: 0, 1.94, 3.87 mg/kg body weight and day, gavage, 18 months recovery period	**1.94 mg/kg body weight and above:** hepatic fibrosis, ascites, bile duct carcinomas, hepatomas	Wilson 1976
dog, mongrel, 2 or 5 ♂, 2 controls	**up to 10 weeks** 0, 2.25, 6.8, 13.6, 27.1 mg 1,2-dimethylhydrazine/kg body weight, 1× weekly; corresponding to: 0, 0.32, 0.97, 1.94, 3.87 mg/kg body weight and day, gavage, 18 months recovery period	**0.32 mg/kg body weight and above:** icterus, liver damage, haemorrhage, hepatic fibrosis; **1.94 mg/kg body weight and above**: mortality	
minipig, 8 or 6 ♂, 4 controls	**up to 10 weeks** 0, 13.6, 27.1 mg 1,2-dimethylhydrazine/kg body weight, 1× weekly; corresponding to: 0, 1.94, 3.87 mg/kg body weight and day, gavage, 18 months recovery period	**1.94 mg/kg body weight and above:** mortality ↑, liver damage, haemorrhage, bile duct proliferation	

#### 5.2.3 Dermal application

There are no data available.

#### 5.2.4 Subcutaneous injection

In a study, 77 rats (no other details) were given a weekly subcutaneous injection of 1,2-dimethylhydrazine of 21 mg/kg body weight (corresponding to 3 mg/kg body weight and day). From the 1^st^ to the 16^th^ week, 4 to 16 animals were killed and examined weekly. By week 5, inflammatory changes of the mucosa in the small and large intestine with crypt abscesses were observed. In addition, an increasing loss of goblet cells and cell proliferation of the mucosal cells of the colon were found. After week 5 up to week 16, adenocarcinomas, papillomas, and signet ring carcinomas appeared in the colon of the treated animals. Adenocarcinomas and mucinous papillomas were detected in the small intestine of the animals from week 8 onwards (Springer et al. 1970).

In the study conducted by Wilson (1976), additional animals were treated subcutaneously. The findings were similar to those following oral administration.

#### 5.2.5 Summary

1,2-Dimethylhydrazine is toxic after repeated exposure, particularly to the liver, bile duct and intestine, and to the heart and kidneys. Thus, at the low dose of 0.32 mg 1,2-dimethylhydrazine/kg body weight and week, icterus, liver damage, haemorrhage and hepatic fibrosis were described in dogs. At 1.94 mg 1,2-dimethylhydrazine/kg body weight and week, rats developed tumours in the colon after 4 and 8 weeks and, after 8 weeks, also in the ear canal. In rats, after weekly doses, inflammation of the intestinal mucosa and cellular changes in the large and small intestines were noted by week 5 of exposure, followed by the development of intestinal tumours. In mice, decreased body weights, decreased relative liver weights and liver damage were observed after 5 months of treatment with 1,2-dimethylhydrazine doses of 0.7 mg/kg body weight and day and above. A NOAEL could not be established.

### 5.3 Local effects on skin and mucous membranes

#### 5.3.1 Skin

1,2-Dimethylhydrazine has a strong alkaline effect and can therefore cause irritation and burns to the skin (Kennedy 2012). In an acute dermal toxicity study in rabbits and guinea pigs, 1,2-dimethylhydrazine was applied once occlusively to the dorsal skin for 24 hours. No skin damage was observed (Rothberg and Cope 1956).

#### 5.3.2 Eyes

Amounts of 3 µl of 1,2-dimethylhydrazine were instilled into the left eye of 2 rabbits. Conjunctivitis and mild inflammation of the eyelids were observed, which had subsided after 5 days (Rothberg and Cope 1956).

### 5.4 Allergenic effects

There are no data available.

### 5.5 Reproductive and developmental toxicity

#### 5.5.1 Fertility

There are no generation studies available.

#### 5.5.2 Developmental toxicity

No evidence of developmental toxicity or teratogenic effects of 1,2-dimethylhydrazine could be derived from a study in 24 pregnant golden hamsters that were given a single gavage dose of 150 mg 1,2-dimethylhydrazine dihydrochloride/kg body weight (67.8 mg 1,2-dimethylhydrazine/kg body weight) on day 12 of gestation. Another 24 golden hamsters served as controls. The incidence of cleft palate was not increased in the offspring. The focus of this study was the activity of intestinal brush border enzymes (lactase, sucrase and alkaline phosphatase) (Schiller et al. 1979). However, as only one single dose was used, the entire duration of organogenesis was not covered; for this reason, the study is not suitable for the assessment of developmental toxicity.

The developmental toxicity of 1,2-dimethylhydrazine was investigated in F344 rats. In this study, 1,2-dimethylhydrazine in saline solution was administered intraperitoneally to 14 to 18 pregnant rats from gestation days 6 to 15. The dose levels were 0, 2, 5 or 10 mg/kg body weight and day. On gestation day 20, the animals were sacrificed and the following parameters were examined: number and positions of implantations, the number of dead and live foetuses, and resorptions. Malformations of the foetuses (external, visceral and skeletal) were additionally examined. Compared with the vehicle control group, body weight gains were reduced in the high dose dams dosed in the early gestational period and in the gestational period after dose administration. This effect on body weights occurred in the middle dose group only in the later time course of treatment. Effects on foetuses were observed only in the high dose group: a reduction in the number of live foetuses per litter and an increase in the number of resorptions per litter. In this group, the number of litters with more than 30% resorptions and malformations was slightly increased (Keller et al. 1984). The authors described that all effects occurred only at 1,2-dimethylhydrazine doses that were toxic also to the dams. As administration was carried out via the intraperitoneal route, a direct effect on the foetuses cannot be excluded. Therefore, the study is not included in the evaluation.

#### 5.5.3 Summary

No developmental or reproductive toxicity studies in accordance with valid test guidelines are available for 1,2-dimethylhydrazine.

### 5.6 Genotoxicity

#### 5.6.1 In vitro

Data from in vitro genotoxicity tests are shown in Table 2.

##### 5.6.1.1 Bacteria and yeasts

In the test for differential killing with Escherichia coli WP2 mutants without metabolic activation, 1,2-dimethyl­hydrazine caused damage to DNA (Poso et al. 1995). As noted in OECD Test Guideline 471 (OECD 2020), the Salmonella typhimurium strains TA97, TA98, TA100, TA1535, TA1537 and TA1538 do not provide reliable evidence of mutagenic effects that are caused by hydrazines. Therefore, the use of the Salmonella typhimurium strain TA102 and Escherichia coli WP2 is recommended for the detection of mutagenic effects induced by this group of substances. The mutagenicity tests available for 1,2-dimethylhylhydrazine confirmed these findings. The studies with the Salmonella strains TA98, TA1535 and TA1538 yielded negative results, the results with the strains TA100 and TA1537 were contradictory. Two positive results were obtained with the strain TA1530. The mutagenicity of 1,2-dimethylhydrazine could be demonstrated with strain TA102 in 2 studies; a third study with negative results used markedly lower concentrations compared with those in the other 2 studies. In Escherichia coli WP2 with and without the plasmid pKM101, 1,2-dimethylhydrazine was mutagenic (Watanabe et al. 1996). Likewise, 1,2-dimethylhydrazine yielded positive results in Escherichia coli ada mutants after activation by chemical oxidation (Sedgwick 1992) and was mutagenic in several Escherichia coli FC mutants (Wei et al. 1996). The substance induced mitotic recombination in yeast cells (Saccharomyces cerevisiae, RS112) (Schiestl 1993).

##### 5.6.1.2 Mammalian cells

###### Indicator tests

DNA damage was examined in various indicator tests with mammalian cells:

1,2-Dimethylhydrazine induced sister chromatid exchanges in CHO cells (MacRae and Stich 1979) and DNA strand breaks were demonstrated via alkaline elution in rat hepatocytes (Sina et al. 1983). The validity of the latter study is questionable, however, because the assessment of cytotoxicity is equivocal. In explanted human bronchial tissue, DNA adducts in the form of N7-methylguanine and O6-methylguanine were detected following exposure to 1,2-dimethylhydrazine (Harris et al. 1977). In in vitro experiments with human explants of non-tumourous colon tissue (Autrup et al. 1980 b) and with human foreskin fibroblasts (Kumari et al. 1985), N7-methylguanine and O6-methylguanine adducts were formed by 1,2-dimethylhydrazine.

In rat and mouse hepatocytes, a UDS (DNA repair synthesis ) assay provided evidence of genotoxicity (Mori et al. 1988).

###### Clastogenicity and mutagenicity tests

Clastogenicity tests, such as chromosomal aberration tests or micronucleus tests, are not available.

In a TK^+/–^ mutation assay with L5178Y mouse lymphoma cells using thymidine excess as a selection agent, induction of small, but not of large, colonies occurred, which may indicate a clastogenic effect. The observed effect was determined at non-cytotoxic concentrations. However, testing for mutation to ouabain, thioguanine, or cytosine arabinoside resistance yielded negative results (Rogers and Back 1981).

No mutagenic effects were detected in an *hprt* mutation assay using V79 hamster cells. The authors explained this by the fact that the S9 mix used for metabolic activation did not contain the enzyme systems required for activation of 1,2-dimethylhydrazine and that the substance is not activated by CYP2E1 (Bronzetti et al. 1996).

DNA sequencing of the whole genome of human induced pluripotent stem (iPS) cells treated with 1,2-dimethylhydrazine (11.6 mM + S9 mix) revealed predominantly single base substitutions such as C:G → T:A (42%) and T:A → C:G (33%). The specific mutation spectrum consisted mainly of the trinucleotide changes NpCpC → NpTpC and NpCpT → NpTpT (N = one of the four bases) and corresponded to a large extent with mutation signature 11 (SBS11) of the COSMIC somatic mutation database, which is associated with exposure to alkylating agents (Kucab et al. 2019; Sanger Institute 2019).

**Tab.2:** In vitro studies of the genotoxicity of 1,2-dimethylhydrazine

End point	Test system	Concentration [µg/plate]^a)^	Effective concentration [µg/plate]^a)^	Cytotoxicity [µg/plate]^a)^	Results		References
					–m. a.	+m. a.	
indicator tests differential killing bacteria	E. coli WP2 trpE56 E. coli CM871 trpE65 uvrA155 recA56 lexA (modified spot test)	WP2: no data. CM871: up to 6010	no data		+	n. t.	Poso et al. 1995
gene mutation bacteria E. coli	E. coli FC215 (wild type), E. coli FC219, E. coli FC321, E. coli FC220, E. coli FC322, E. coli FC221, E. coli FC325, E. coli FC222, E. coli FC323, E. coli FC223, E. coli FC326 (lacZ ada-deficient, ogt-deficient or MTase-deficient strains)	0, 1, 5 µg/ml	–	no data	–	n. t.	Wei et al. 1996
	E. coli FC218 (GC→AT mutation necessary for reversion, MTase-deficient)	0, 1, 5 µg/ml	1 µg/ml	no data	+	n. t.	
	E. coli ada mutants, chemical oxidation as activation mechanism	2 mM	2 mM	+	–	+	Sedgwick 1992
	E. coli WP2/pKM101, E. coli WP2uvrA/pKM101 (plate incorporation test)	0, 313, 625, 1250, 2500, 5000	≥ 2500	no data	+	n. t.	Watanabe et al. 1996
	E. coli WP2 uvrA trp (spot test and modified pre-incubation test)	0, 30, 60, 120 µg/ml	–	> 120 µg/ml	–	–	von Wright and Tikkanen 1980
gene mutation S. typhimurium	S. typhimurium TA100 (plate incorporation test)	0, 60, 120, 180, 902, 1803, 3005	–	> 3005	–	–	von Wright and Tikkanen 1980
	S. typhimurium TA100 (plate incorporation test)	0, 565, 1130, 2260, 4520, 9040	≥ 1130	≥ 9040	+	+	Parodi et al. 1981
	S. typhimurium TA1535 (plate incorporation test)	0, 565, 1130, 2260, 4520, 9040	–	no data	–	–	
	S. typhimurium TA1537 (plate incorporation test)	0, 565, 1130, 2260, 4520, 9040	–	no data	–	–	
	S. typhimurium TA1538 (plate incorporation test)	0, 565, 1130, 2260, 4520, 9040	–	no data	–	–	
	S. typhimurium TA98 (plate incorporation test)	up to solubility limit/toxicity limit	–	no data	–	–	De Flora 1981
	S. typhimurium TA100 (plate incorporation test)	up to solubility limit/toxicity limit	≥ 600	no data	+	+	
	S. typhimurium TA1535 (plate incorporation test)	up to solubility limit/toxicity limit	–	no data	–	–	
	S. typhimurium TA1537 (plate incorporation test)	up to solubility limit/toxicity limit	–	no data	–	–	
	S. typhimurium TA1538 (plate incorporation test)	up to solubility limit/toxicity limit	–	no data	–	–	
	S. typhimurium TA100 (pre-incubation test)	0, 2.5, 5.0, 10.0, 25 mM	–	no toxicity up to 25 mM	no data	–	Malaveille et al. 1983
	S. typhimurium TA1530 (pre-incubation test)	0, 2.5, 5.0, 10.0, 25 mM 0, 0.1, 0.5, 1 mM	0.1 mM	≥ 10 mM	+	+	
	S. typhimurium TA1535 (pre-incubation test)	0, 2.5, 5.0, 10.0, 25 mM	–	no data	no data	–	
	S. typhimurium TA100 (pre-incubation test)	0–601 growth period before treatment 11 hours	–	no data	–	–	Matsushita et al. 1993
	S. typhimurium TA102 (pre-incubation test)	0–601 growth period before treatment 5, 7 or 11 hours	no data + after 5 hours growth period	no data	+	–	
	S. typhimurium TA102 (pre-incubation test)	0, 75, 150, 300	–	no data	–	n. t.	Poso et al. 1995
	S. typhimurium TA1535 (pre-incubation test)	0, 100, 200, 500, 1000	200	1000	(+)	–	Rogan et al. 1982
	S. typhimurium TA1537 (pre-incubation test)	0, 100, 200, 500, 1000	100	1000	(+)	(+)	
	S. typhimurium TA102 (plate incorporation test)	0, 313, 625, 1250, 2500, 5000	≥ 2500	–	(+)	n. t.	Watanabe et al. 1996
	S. typhimurium TA2638 (plate incorporation test)	0, 313, 625, 1250, 2500, 5000	≥ 2500	–	+	n. t.	
	S. typhimurium TA98 (plate incorporation test)	no data	–	no data	–	–	Wilpart et al. 1983
	S. typhimurium TA100 (plate incorporation test)	no data	–	no data	–	–	
	S. typhimurium TA100 (plate incorporation test)	0, 80, 100, 200, 300 (1.2-DMH) + 100, 300 litocholic acid or deoxycholic acid	80	no data	+	+	
	S. typhimurium TA1530 (plate incorporation test)	100 in DMSO	–	no data	–	–	
	S. typhimurium TA1530 (plate incorporation test)	100 in ethanol	100	no data	+	–	
	S. typhimurium TA1538 (plate incorporation test)	no data	–	no data	–	–	
	S. typhimurium YG7108, MTase-deficient (plate incorporation test)	0–200 µg/ml	10 µg/ml	no data	+	+	Wei et al. 1996
	S. typhimurium YG7108 with PYG616 plasmid, Mtase proficient (plate incorporation test)	0–200 µg/ml	200 µg/ml	no data	+	+	
	S. typhimurium TA1535 (spot test)	0–200 µg/ml	–	no data	–	–	
mitotic recombination in yeast	S. cerevisiae RS112	no other details	no data	no data	+	n. t.	Schiestl 1993
indicator tests mammalian cells	sister chromatid exchange, CHO cells (hamster), exposure for 3 hours or 24 hours	0, 0.063, 0.13, 0.25, 0.5, 1, 2 mM	1 mM after 3 h, 0.063 mM after 24 h	0.5 mM after 24 h	+	n. t.	MacRae and Stich 1979
	DNA strand breaks, alkaline elution, rat hepatocytes	0, 0.03, 0.3, 3 mM	0.3 mM	determination of cytotoxicity not valid	+	–	Sina et al. 1983
	covalent DNA binding, explanted human bronchial tissue (1 patient)	1.29 mM	1.29 mM	no data	+ (N7-, O6-MeGua)	n. t.	Harris et al. 1977
	covalent DNA binding, human preputial fibroblasts, exposure for 6 hours	0.5 mM	no data	no data	+ (N7-, O6-MeGua, 3-MeAde)	n. t.	Kumari et al. 1985
	covalent DNA binding, explanted human colon tissue, exposure for 24 hours	0.1 mM	no data	no data	+	n. t.	Autrup et al. 1980 a
	covalent DNA binding, explanted human colon tissue, exposure for 24 hours	0.1 mM	no data	no data	+ (N7-, O6-MeGua)	n. t.	Autrup et al. 1980 b
	covalent DNA binding, rat, colon tissue, exposure for 24 hours	0.1 mM	no data	no data	+ (N7-, O6-MeGua)	n. t.	Autrup et al. 1980 b
	UDS, rat hepatocytes (ACI)	0, 0.45–45.2 mM	1 mM	not cytotoxic in tested range	+	n. t.	Mori et al. 1988
	UDS, mouse hepatocytes (C3H/HeN)	0, 0.45–45.2 mM	1 mM	not cytotoxic in tested range	+	n. t.	
gene mutation mammalian cells	TK^+/–^ test, mouse lymphoma cells (L5178Y), selection agent: thymidine excess	0, 0.1, 1, 2.5, 5 mM	about 1 mM	≤ 20% cytotoxicity up to 5 mM	+ (small colonies)	n. t.	Rogers and Back 1981
	mutation to ouabain, thioguanine or cytosine arabinoside resistance, mouse lymphoma cells (L5178Y)	0, 0.1, 1, 2.5, 5 mM	–	≥ 20 mM	–	n. t.	
	hprt-test, hamster lung fibroblasts (V79)	0, 1–20 mM	–	no data	–	–	Bronzetti et al. 1996
	genome sequencing, human induced pluripotent strain cells (iPS)	–m. a.: 9 mM (24 hours) +m. a.: 11.6 mM (3 hours)	+m. a.: 11.6 mM (24 hours)	40%–60%; IC_50_: –m. a: 8.3 (24 hours) +m. a.: 11.2 mM (3 hours)	–	+	Kucab et al. 2019

a) unless indicated otherwise

–: negative result; +: positive result; (+): weakly positive; CHO: Chinese hamster ovary; 1.2-DMH: 1,2-dimethylhydrazine; DMSO: dimethylsulf­oxide; E. coli: Escherichia coli; IC: inhibition concentration; m. a.: metabolic activation; MeAde: methyladenine; MeGua: methylguanine; MTase: methyltransferase; n. t.: not tested; S. cerevisiae: Saccharomyces cerevisiae; S. typhimurium: Salmonella typhimurium; UDS: DNA repair synthesis

#### 5.6.2 In vivo

The data from the in vivo genotoxicity tests are shown in Table 3.

##### 5.6.2.1 Drosophila

An eye mosaic test in Drosophila melanogaster revealed 1,2-dimethylhydrazine to be genotoxic in 2 genotypes at doses of 1 or 5 mM (Rodriguez-Arnaiz 1997). In the SLRL (sex-linked recessive lethal mutation) assay in Drosophila melanogaster, flies were fed 1,2-dimethylhydrazine with and without the addition of various inhibitors of metabolizing enzyme systems (1-phenylimidazole, iproniazid, *N*,*N*-dimethylbenzylamine). No genotoxic effects were observed with or without enzyme inhibitors (Zijlstra and Vogel 1988).

##### 5.6.2.2 Bacteria in vivo–ex vivo

Host-mediated assays revealed a mutagenic effect of 1,2-dimethylhydrazine after intraperitoneal or oral administration (Kerklaan et al. 1986; Moriya et al. 1979; von Wright and Tikkanen 1980; Zeilmaker et al. 1991). Analysis of the *lacI* gene revealed that 91% of the induced mutations were single base substitutions of the G:C → A:T type (Zeilmaker et al. 1991).

##### 5.6.2.3 Mammals

###### Indicator tests

Sister chromatid exchange was induced in intestinal epithelial cells of C57BL/6J mice after single intraperitoneal injections of 5, 10 or 20 mg/kg body weight, but not in bone marrow cells at 0, 10, 20, 30 or 40 mg/kg body weight (Couch et al. 1986). Another study revealed sister chromatid exchange in bone marrow cells, liver cells, kidney cells and alveolar macrophages from B6D2F mice at 4.1 up to 19.3 mg/kg body weight (Neft and Conner 1989).

DNA strand breaks were detected in rats and mice of different strains (Table 3). The methods used were alkaline elution and the comet assay. Treatment was either by gavage, subcutaneously or intraperitoneally; mainly single doses were used. The liver, colon, stomach, lungs and kidneys were examined, with the strongest effect in the liver.

In tests with rats and mice of different strains, covalent DNA binding by methylation was detected. The DNA adducts detected were primarily N7-methylguanine and O6-methylguanine and formed after subcutaneous, oral and intraperitoneal administration. The O6-methylguanine adducts were found primarily in the liver and colon, and the N7-methylguanine adducts formed in the liver, intestine, kidneys and lungs of the animals. In addition to O6-methyl­guanine also O4-methylthymidine was detected, but in much lower amounts (O’Toole et al. 1993; Richardson et al. 1985), and C8-methylguanine (Netto et al. 1992).

Covalent binding of methyl groups to DNA in germ cells of BD-VI rats was detected (Likhachev et al. 1977). Since the route of administration was subcutaneous, accessibility of the germ cells can be concluded.

In a UDS assay, evidence of DNA damage caused by 1,2-dimethylhydrazine was demonstrated in the liver (20 mg/kg body weight, single oral dose) but not in the kidneys of the rat (50 mg/kg body weight, single intraperitoneal dose) (Mirsalis et al. 1982; Tyson and Mirsalis 1985). In rats given single gavage doses of 20 mg 1,2-dimethylhydrazine/kg body weight, the UDS test yielded a positive result in hepatocytes (Beije and Olsson 1990).

###### Clastogenicity and mutagenicity tests

1,2-Dimethylhydrazine induced nuclear abnormalities (micronuclei, pyknotic nuclei, fragmented nuclei) in the ­colon of C57BL/6J mice given a single dose of 0, 12, 25 or 50 mg/kg body weight (Wargovich et al. 1983). Micronucleus tests in mice and rats of different strains yielded positive results for intestinal tissue after oral and intraperitoneal administration (Goldberg et al. 1983, 1991; Zhurkov et al. 1996). Likewise, positive results were recorded for the liver, gastrointestinal tract, blood and bone marrow after oral administration (Albanese et al. 1988; Chikura et al. 2016; Criswell et al. 2003; Goldberg et al. 1991; Meli and Seeberg 1990; Morrison and Ashby 1995; Suzuki et al. 2008; Tinwell et al. 1990; Vanhauwaert et al. 2001; Wakata et al. 1998). In the bone marrow, the results were in some cases contradict­ory. This may be explained by an insufficient exposure time to reactive metabolites or the parent substance at the target site. If the number of examined cells is too low (< 2000 polychromatic erythrocytes), this can likewise produce a false-negative result.

Although none of the micronucleus tests allows the differentiation between aneugenic and clastogenic effects, in the light of all available data, a clastogenic effect has to be assumed. Details of the studies are shown in Table 3.

No mutagenic effects were detected in a pig-a/PIGRET assay in SD rats (dose levels 0, 25, 50 and 100 mg/kg body weight). However, this was explained by a possible delay in the cell cycle and cell proliferation caused by the substance, which may have prevented expression of the mutagenic phenotype during the duration of the experiment (Chikura et al. 2016).

In gene mutation studies (Dlb-1 locus assay), the substance was not found to have a mutagenic effect in intestinal cells of the mouse (C57BL/6J × SWR F1) after a single intraperitoneal dose, unlike after 10 doses of 10, 20 or 30 mg/kg body weight (Tao and Heddle 1994). Accordingly, in mouse intestinal cells (C57BL/6J × SWR F1), a mutagenic effect was detected in the Dlb-1 locus assay after multiple, but not after single, subcutaneous doses (Winton et al. 1990). In CD-1 mice, a mutagenic effect of 1,2-dimethylhydrazine was observed in the liver at the *trp53* locus after 2 oral doses of 20 mg/kg body weight (Jenkins et al. 1997).

**Tab.3:** In vivo studies of the genotoxicity of 1,2-dimethylhydrazine

Test system		Dose	Results	Remarks	References
**Drosophila melanogaster**					
somatic mutations and reciprocal recombinations	eye mosaic test, Drosophila melanogaster (genotypes ST, HK, HG)	0, 0.5, 1.0, 5.0 mM	(+)	1 mM (ST) or 5 mM (HG), ST at 5 mM decrease in survival	Rodriguez-Arnaiz 1997
SLRL sex-linked recessive lethal mutations	Drosophila melanogaster	0, 3, 5 mM, in the diet or injected	–	co-treatment or pre-treatment with enzyme inhibitors without effect on results	Zijlstra and Vogel 1988
**In-vivo–ex-vivo**					
host-mediated assay	mouse (ICR), ♂ (no other details), Salmonella typhimurium TA G46	0–60 mg/kg body weight, gavage	+	dose-dependent at 45 mg/kg body weight and above	Moriya et al. 1979
	mouse (NMRI), 4 ♂ per group, Salmonella typhimurium TA1950	0, 100 mg/kg body weight, gavage	+		von Wright and Tikkanen 1980
	mouse (Swiss), 3 ♀ per group, Escherichia coli 343/113	0, 60 mg/kg body weight, intraperitoneal	+		Kerklaan et al. 1986
	mouse (Swiss), 2–4 ♀ per group, Escherichia coli NR8090	0, 6, 30 mg/kg body weight, intraperitoneal	+	+ at 30 mg/kg body weight; *lacI *mutations	Zeilmaker et al. 1991
**Mammals**					
indicator tests	sister chromatid exchange, mouse (C57BL/6J), 2–5 ♀ per group, colon, sampling 26–73 hours after administration, BrdU administration 48 hours before killing of the animals	0, 20 mg/kg body weight, single, intraperitoneal	+	+ 26–62 hours after administration	Couch et al. 1986
	sister chromatid exchange, mouse (C57BL/6J), 3–4 ♀ per group, colon, sampling 46 hours after administration	0, 5, 10, 20 mg/kg body weight, single, intraperitoneal	+	+ at 10 mg/kg body weight and above, dose-dependent	
	sister chromatid exchange, mouse (C57BL/6J), 4–5 ♀ per group, bone marrow, sampling 14 hours after administration	0, 10, 20, 30, 40 mg/kg body weight, single, intraperitoneal	–		
	sister chromatid exchange, mouse (B6D2F1), 3–16 ♂ per group, bone marrow, liver, kidneys, alveolar macrophages	0, 4.1, 4.88, 7.3, 9.5, 19.3 mg/kg body weight, single, intraperitoneal	+	2 groups per dose: intact liver / partial hepatectomy	Neft and Conner 1989
	sister chromatid exchange, mouse (B6D2F1), 3–7 ♂ per group, bone marrow, liver, kidneys, alveolar macrophages	0, 4.1 mg/kg body weight, once per week, 10 weeks, intraperitoneal	+	2 groups per dose: intact liver / partial hepatectomy	
	DNA strand breaks, alkaline elution, mouse (BALB/c, C57BL/6, Swiss), ♂ (number not given), liver, colon, stomach, lungs, kidneys, sampling 4–24 hours after administration	0, 12.5–200 mg/kg body weight, single, gavage or subcutaneously	+	liver, colon, stomach, lungs, kidneys, dose-dependent DNA damage, particularly in liver; in colon: Swiss > BALB/c > C57BL/6	Brambilla et al. 1978
	DNA strand breaks, alkaline elution, mouse (Swiss albino), 6–9 ♂ per group, lungs, liver, sampling 1 or 6 hours after administration	0, 85.9 mg/kg body weight, single, intraperitoneal	+	DNA damage: liver > lungs	Parodi et al. 1981
	DNA strand breaks, alkaline elution, mouse (Swiss albino), 6 ♂ per group, lungs, liver, sampling 6 hours after final dose	0, 14.4 mg/kg body weight and day, 5 days, intraperitoneal	+	DNA damage: liver > lungs	
	DNA strand breaks, alkaline elution, mouse (AKR/J, DBA/2, CD1, C57BL/6N, SWR/J, B6D2F1), 5 animals per group (sex not specified), liver, kidneys, colon, sampling 4 hours after administration	0, 50 mg/kg body weight, single, intraperitoneal	+	in all strains: DNA damage: liver > kidneys >> colon	Bolognesi and Boffa 1986
	DNA strand breaks, alkaline elution, rat (CD), 6–8 ♀ per group (controls 18), liver, sampling 21 hours after administration	0.0014, 0.0045, 0.014, 0.045, 0.14, 0.45, 1.0, 2.0, 4.5, 10, 20, 45, 135 mg/kg body weight, single, gavage	+	+ at 1 mg/kg body weight and above	Kitchin and Brown 1996
	DNA damage, comet assay, rat, 6 animals per group (sex not specified), colon, sampling 25 weeks after final dose	0, 20 mg/kg body weight, once per week, 15 weeks, subcutaneous	+	documentation inadequate	Kumar et al. 2010
	DNA damage, comet assay, rat (SD), 6 ♂ per group, liver, sampling 3 and 24 hours after administration	0, 12.5, 25, 50 mg/kg body weight, single, gavage	+	12.5 mg/kg body weight and above	Rothfuss et al. 2010
	DNA damage, comet assay, rat (SD), 6 ♂ per group, liver	0, 1.25, 2.5, 5 mg/kg body weight and day, 29 days, gavage	+	+ at 1.25 mg/kg body weight and above, histopathological changes at 1.25 mg/kg body weight and above, MTD: 5 mg/kg body weight and day	
	DNA damage, comet assay, rat (SD), 6 ♂ per group, liver, sampling 3 and 24 hours after administration	0, 25, 50, 100 mg/kg body weight, single, gavage		cannot be evaluated, as too many hedgehog cells (= apoptosis)	Chikura et al. 2016
		0, 4, 10, 25 mg/kg body weight, single, gavage	+	4 mg/kg body weight and above, dose-dependent increase 3 hours after administration, effect slight 24 hours after administration, no increase between 10 and 25 mg/kg body weight	
	covalent DNA and RNA binding, mouse (NMRI), 12 ♀, liver, colon, kidneys, lungs, small intestine, spleen, sampling 6 hours after administration	15 mg/kg body weight, single, subcutaneous	+	N7-methylguanine: liver >> colon, spleen, kidneys > lungs, small intestine	Hawks and Magee 1974
	covalent DNA and RNA binding, rat (Wistar), 5 ♂, liver, colon, kidneys, lungs, small intestine, spleen sampling 6 hours after administration	200 mg/kg body weight, single, subcutaneous	+	N7-methylguanine: liver > colon > kidneys	
	covalent DNA binding, rat (F344), 3–6 ♂ per group, hepatocytes, sampling after 12, 24, 48, 72, 120 hours	20 mg/kg body weight, single, subcutaneous	+	O6-methylguanine/O4-methylthymidine, 12 and 24 hours after administration about 100:1	Richardson et al. 1985
	covalent DNA binding, rat (F344), 5 ♂ per group, liver, sampling 12, 24, 36, 48 hours after administration	0, 1.7 or 20 mg/kg body weight (contradictory data), single, subcutaneous	+	N7-methylguanine, O4-methylthymidine, O6-methylguanine	O’Toole et al. 1993
	covalent DNA binding, rat (BD-VI), 5 ♂, liver, colon, kidneys, testes, lungs, small intestine, duodenum, sampling after 3 hours	300 mg/kg body weight, single, subcutaneous	+	O6-methylguanine (only in liver and colon), N7-methylguanine liver >> colon > kidneys > testes > lungs and small intestine	Likhachev et al. 1977
	covalent DNA binding, rat (CFN-Wistar), 4 ♂ per group, colon	0, 60 mg/kg body weight, single, intraperitoneal	+	no specification of DNA binding, additional methylation of proteins (including histone)	Boffa et al. 1982
	covalent DNA binding, mouse (ICR/Ha), 10 ♂ per group, colon, sampling 6, 14 and 40 hours after administration	0, 20 mg/kg body weight, single, intraperitoneal	+	N7-methylguanine > O6-methylguanine	James and Autrup 1983
	covalent DNA binding, mouse (ICR/Ha) or (C57BL/Ha), 2–4 ♂, 2–4 ♀ per group, colon, sampling 6, 14, 40 and 96 hours after administration	20 mg/kg body weight, single, intraperitoneal	+	N7-methylguanine > O6-methylguanine, no difference between strains	
	covalent DNA binding, mouse (ICR/Ha) or (C57BL/Ha), 2–4 ♂, 2–4 ♀ per group, colon, sampling 6, 14, 40 and 96 hours after administration	20 mg/kg body weight, once per week, 5 weeks, intraperitoneal	+	N7-methylguanine > O6-methylguanine, O6-methylguanine: ICR/Ha > C57BL/Ha	
	covalent DNA binding, rat (Wistar), 3–4 ♂ per group, colon, liver, sampling 1–24 hours after administration	0, 20 mg/kg body weight, single, subcutaneous	+	N7-methylguanine, O6-methylguanine	Tacchi-Bedford et al. 1988
	covalent DNA binding, rat (F344), 3 ♂ per group, liver	1.7–2.4 mg/kg body weight and day, 1, 2, 3, 4, 6, 8, 12, 16, 28 days, drinking water	+	N7-methylguanine >> O6-methylguanine	Bedell et al. 1982
	covalent DNA binding or DNA repair, rat (F344), 3 ♂ per group, liver	1.7–2.4 mg/kg body weight and day, 2, 4, 8, 16 days, drinking water	+	N7-methylguanine > O6-methylguanine	Lewis and Swenberg 1983
	covalent DNA binding, rat (SD and Wistar), no other details, colon, liver	0, 300 mg/kg body weight, single, intraperitoneal	+	C8-methylguanine, N7-methylguanine, O6-methylguanine	Netto et al. 1992
	UDS test, rat (F344), 3 ♂ per group, kidneys, sampling 2 and 12 hours after administration	0, 50 mg/kg body weight, single, intraperitoneal	–	no data for cytotoxicity	Tyson and Mirsalis 1985
	UDS test, rat (F344) 3 ♂ per group, liver, sampling 12 hours after administration	0, 20 mg/kg body weight, single, gavage	+		Mirsalis et al. 1982
	UDS test, rat, no other details	0, 20 mg/kg body weight, gavage	+		Beije and Olsson 1990
**Soma cells**					
nuclear anomalies (micronuclei, pyknotic cell nuclei, fragmented cell nuclei)	mouse (C57BL/6J), 3 ♂, 3 ♀ per group, colon, sampling after 24 hours	0, 12, 25, 50 mg/kg body weight, single, gavage	+	12 mg/kg body weight and above	Wargovich et al. 1983
micronucleus test	mouse (C57BL/6J), 3 ♀, 5 ♂ per group, colon, bone marrow, sampling 24, 48, 72 hours after administration	0, 15 mg/kg body weight, single, intraperitoneal	+	1/2 of LD_50_; apart from micronuclei, colon cells displayed apoptosis, pyknotic cell nuclei and karyolysis, + colon after 24 hours, – bone marrow, 500 cells per animal counted	Goldberg et al. 1983
	mouse (C57BL/6J), 5 ♂ per group, colon, bone marrow, sampling 24 hours after administration	0, 7.5, 15, 30, 45 mg/kg body weight, single, intraperitoneal	+	+ colon at 15 mg/kg body weight and above, no data for cytotoxicity, – bone marrow, not cytotoxic in bone marrow	
	mouse (CBA), 4–7 ♂ per group, bone marrow, sampling 24 hours after administration	0, 10, 50 mg/kg body weight, single, gavage	+	10 mg/kg body weight and above, no data for cytotoxicity	Ashby and Mirkova 1987
	mouse (CCBF1), 5 ♂ per group, bone marrow, sampling 24 and 48 hours after administration	0, 50 mg/kg body weight, single, gavage	+	50 mg/kg body weight corresponding to 1/2 LD_50_, no data for cytotoxicity	Albanese et al. 1988
	mouse (C57BL/6J), 6 ♂ per group (2 experiments), bone marrow, sampling 24 hours after administration	0, 50 mg/kg body weight, single, gavage	+	no data for cytotoxicity; only one time point evaluated	
	rat (Alderley Park), 5 ♂ per group, bone marrow, sampling 24 and 28 hours after administration	0, 50 mg/kg body weight, single, gavage	–		
	rat (Alderley Park), 6 or 10 ♂ per group (2 experiments), bone marrow, sampling 24 hours after administration	0, 80 mg/kg body weight, single, gavage	–	only one time point evaluated	
	mouse (CBA), 8 ♂ per group, bone marrow, sampling 24 hours after administration	0, 25, 45 mg/kg body weight, single, gavage	+	+ at 25 mg/kg body weight, cytotoxicity at 45 mg/kg body weight	Morrison and Ashby 1995
	mouse (CBA), 10 ♂ per group, bone marrow, sampling 24 hours after administration	0, 25, 45 mg/kg body weight, single, gavage	+	+ at 25 mg/kg body weight and above, cytotoxicity at 45 mg/kg body weight, 2000 PCE/animal counted	
	mouse (CBAB6F1), 5–6 ♂, 6 ♀ per group, colon, forestomach, stomach, bone marrow, sampling 24 hours after final dose	0, 0.72, 3.6,18 mg/kg body weight and day, 3 days gavage	+	+ colon only 1000 PCE/animal counted, no data for cytotoxicity	Zhurkov et al. 1996
	mouse (CBAB6F1), 5–7 ♂, 5–6 ♀ per group, liver, 1 hour after final dose partial hepatectomy, 4 days later sampling	0, 0.72, 3.6, 18 mg/kg body weight and day, 13 days, gavage	+		
	rat (no other details), 6 ♂, 6 ♀ per group, forestomach, stomach, duodenum, small intestine, colon, bone marrow, sampling 24 hours after final dose	0 3.32, 16.6, 33.2 mg/kg body weight and day, 3 days, gavage	+	+ colon only 1000 PCE/animal counted, no data for cytotoxicity	
	rat (Crl:(WI)BR), 5 ♂ per group, bone marrow, sampling after 24 and 48 hours	0, 50, 100, 150 mg/kg body weight, single, oral	(+)	analysis: manually and using flow through cytometry; leukocytes and thrombocytes in the blood significantly ↓	Criswell et al. 2003
	rat (SD), 5–6 ♂ per group, bone marrow, blood, sampling after 24 hours	0, 25, 50, 100 mg/kg body weight, twice: 0, 21 hours gavage	+		Chikura et al. 2016
	rat (F344), 4 ♂ per group, 4 weeks old, liver, sampling 3, 4, 5 days after administration	0, 45.2, 90.5 mg/kg body weight, single, gavage	+	MI significantly ↓ at 45.2 mg/kg body weight after 3 days and at 90.5 mg/kg body weight after 3, 4 and 5 days mortality at 200 mg/kg body weight from day 4 after administration	Suzuki et al. 2008
	rat (F344), 4–5 ♂ per group, blood, bone marrow, sampling: bone marrow 24 hours after final dose, peripheral blood 24 hours before 1^st^ dose and 24 hours after 2^nd^, 3^rd^, 4^th^, 7^th^, 14^th^, 21^st^ and 28^th^ dose	0, 5, 15, 50 mg/kg body weight and day, 28 days, gavage^a)^	–	– peripheral blood, bone marrow, 28 days, mortality at 5 mg/kg body weight and above: eosinophilic and vacuolar degeneration in the liver at 15 mg/kg body weight and above: all animals died, extramedullary haematopoiesis ↑ in spleen and liver, haematopoietic cells ↑ in bone marrow, severe liver damage (including necrosis, invasive haemorrhages, degeneration), atrophy of the white pulpa of the spleen	Hamada et al. 2001
	mouse (CD1), bone marrow, colon, preliminary study: 2 ♂, 2 ♀ per group, sampling 12, 18, 24, 36, 48, 72 hours after administration, main study (1.2-DMH as positive control): 5 ♂ per group, sampling 24 and 48 hours after administration	preliminary study: 0, 20, 30 mg/kg body weight, single, main study: 0, 20 mg/kg body weight, single, gavage	+	+ colon at 20 mg/kg body weight and above, preliminary study: maximum induction 36 hours after administration, dose-dependent increase in number of apoptotic cells, bone marrow: –, no cytotoxicity	Vanhauwaert et al. 2001
	rat (SD), 6 ♂ per group, bone marrow, peripheral blood, blood sampling on days 0, 4, 15, 29, bone marrow investigated after 29 days	0, 1.25, 2.5, 5 mg/kg body weight and day, 28 days, gavage	–	cytotoxicity at 5 mg/kg body weight (peripheral blood from day 15 onwards), liver toxicity at 1.25 mg/kg body weight and above, MTD: 5 mg/kg body weight and day (body weight gains ↓)	Rothfuss et al. 2010
	rat (TUC(SD)spf), 3 ♂ per group, bone marrow	0, 50, 100, 200 or 0, 20, 32, 50 mg/kg body weight, single, intraperitoneal	–	only 300 PCE/animal evaluated	Trzos et al. 1978
	mouse (CBA), 4–6 ♂ per group, bone marrow, sampling 24 hours after administration	0, 11.2, 53.7 mg/kg body weight, single, gavage	+	2000 PCE/animal evaluated	Tinwell et al. 1990
	mouse (CBA), 4–6 ♂ per group, bone marrow, sampling 24 hours after final dose	0, 4.5, 11.2, 17.9 mg/kg body weight and day, 3 days	+	2000 PCE/animal evaluated + at 17.9 mg/kg body weight and above	
		0, 11.2 mg/kg body weight and day, 2 days, gavage			
	mouse (CD1), 5 ♂, 5 ♀ per group, bone marrow, sampling 24 hours after administration	0; 10.4; 20.7; 41.4 mg/kg body weight, single, gavage	+	1000 PCE/animal evaluated + at 10.4 mg/kg body weight and above (♀), 41.4 mg/kg body weight (♂), no cytotoxicity	Meli and Seeberg 1990
	mouse (CD1), 5 ♂, 5 ♀ per group, bone marrow, sampling 24 hours after final dose	0, 3.45, 6.9, 13.8 mg/kg body weight and day, 3 days, gavage	–	1000 PCE/animal evaluated	
	mouse (CD1), 5 ♂, 5 ♀ per group, bone marrow, sampling 24 hours after final dose	0, 13.8, 15.5, 17.2 mg/kg body weight and day, 3 days, gavage	+	1000 PCE/animal evaluated MN significantly ↑ (♂: at 15.5 mg/kg body weight; ♀: at 17.2 mg/kg body weight; ♂ and ♀: at 15.5 mg/kg body weight and above), cytotoxicity and mortality ↑ at 17.2 mg/kg body weight	
	mouse (C57BL/6J), 7 ♂ per group, bone marrow, colon, sampling 24 hours after administration	0, 10, 20, 50 mg/kg body weight, single, intraperitoneal	+	1000 PCE/animal evaluated bone marrow: MN significantly ↑ at 50 mg/kg body weight, no cytotoxicity, colon: MN significantly ↑ at and above 10 mg/kg body weight, C57BL/6J strain more sensitive	Goldberg et al. 1991
	mouse (C57BL/6J), 7 ♂ per group, bone marrow, colon, sampling 24 hours after administration	0, 10, 20, 50 mg/kg body weight, single, gavage	+	1000 PCE/animal evaluated bone marrow: –, no cytotoxicity, colon: MN significantly ↑ at and above 10 mg/kg body weight, C57BL/6J strain more sensitive	
	mouse (CBA), 11 ♂ per group, bone marrow, colon, sampling 24 hours after administration	0, 10, 20, 50 mg/kg body weight, single, intraperitoneal	+	1000 PCE/animal evaluated bone marrow: MN significantly ↑ at and above 20 mg/kg body weight, no cytotoxicity, CBA strain more sensitive than C57BL/6J, colon: MN significantly ↑ at and above 10 mg/kg body weight	
	mouse (CBA), 16 ♂ per group, bone marrow, colon, sampling 24 hours after administration	0, 10, 20, 50 mg/kg body weight, single, gavage	+	1000 PCE/animal evaluated bone marrow: MN significantly ↑ at 50 mg/kg body weight, no cytotoxicity, colon: MN significantly ↑ at and above 10 mg/kg body weight, CBA strain more sensitive than C57BL/6J	
	rat (SD), 5–6 ♂ per group, bone marrow, sampling 24 hours after administration	0, 25, 50, 100 mg/kg body weight, single, gavage	+	at and above 25 mg/kg body weight; cytotoxic at 100 mg/kg body weight	Chikura et al. 2016
gene mutation	Dlb1 locus, mouse [(C57BL/6J × SWR)F1], 4–6 ♀ per group, small intestine	0, 1, 7.5, 15 mg/kg body weight, single, subcutaneous	–		Winton et al. 1990
	Dlb1 locus, mouse [(C57BL/6J × SWR)F1], 6 ♀ per group, small intestine, sampling 2 or 12 weeks after final dose	0, 20 mg/kg body weight, once per week, 10 weeks, subcutaneous	+		
	Dlb1 locus, mouse (C57BL/6J) (homozygous for DIb1 locus), 3 ♀ per group, small intestine, sampling 2 weeks after final dose	0, 20 mg/kg body weight, once per week, 10 weeks, subcutaneous	–		
	Dlb1 locus, mouse [(C57BL/6J × SWR)F1], 5 ♂, ♀ per group, small intestine, sampling 2–8 weeks after administration	0, 10, 20, 30 mg/kg body weight, single, intraperitoneal	–		Tao and Heddle 1994
	Dlb1 locus, mouse [(C57BL/6J × SWR)F1], 5 ♂, 5 ♀ per group, small intestine, sampling 2–8 weeks after final dose	0, 10, 20, 30 mg/kg body weight, once per week, 10 weeks, intraperitoneal	+	at 10 mg/kg body weight and above	
	trp53, (exons 4 and 5, intron 6), restriction site mutation (RSM) test, mouse (CD-1), 4 ♂ per group, liver, sampling 24 hours after final dose	0, 20 mg/kg body weight and day, 2 days, gavage	+	mutation rate: intron 6 >> exon 4 > exon 5, mainly G:C → A:T, no data for cytotoxicity	Jenkins et al. 1997
	Pig-a, PIGRET, rat (SD), 6 ♂ per group, sampling 1, 2 and 4 weeks after administration	0, 25, 50, 100 mg/kg body weight, single, gavage	–	PIGRET at 50 mg/kg body weight significantly + due to outliers, up to 100 mg/kg body weight not cytotoxic	Chikura et al. 2016

–: negative result; +: positive result; (+): result unclear; 1.2-DMH: 1,2-dimethylhydrazine; MI: mitotic index; MN: micronuclei; MTD: maximum tolerated dose; PCE: polychromatic erythrocytes; UDS: DNA repair synthesis

a) data given for 1,2-dimethylhydrazine dihydrochloride, details of the study unclear

#### 5.6.3 Accessibility of the germ cells

To investigate the inhibition of testicular DNA synthesis by 1,2-dimethylhydrazine, male mice were given gavage doses of 200 mg/kg body weight. The incorporation of ^3^H-thymidine into testicular DNA of mice (as a measure of DNA synthesis) amounted to 71.3% of the control value (Seiler 1977).

Accessibility of the germ cells has been demonstrated for 1,2-dimethylhydrazine by the covalent binding of methyl groups to testicular DNA of mice after subcutaneous injection (Likhachev et al. 1977).

#### 5.6.4 Summary

Even though the numerous studies that are available are older and were not carried out according to current test guidelines, it is possible to draw the following conclusions with respect to genotoxicity:

1,2-Dimethyhydrazine induced gene mutations in Escherichia coli strains. In the Salmonella mutagenicity test, the substance was mutagenic in the strain TA102; contradictory results were obtained in the other strains. There is evidence of mutagenicity in mammalian cells in vitro. Numerous indicator tests in vitro and in vivo indicate DNA-damaging effects: sister chromatid exchange, DNA strand breaks and UDS were induced and the DNA adducts N7-methyl­guanine and O6-methylguanine were detected in vitro. In vivo in the liver of rats additionally C8-methylguanine and O4-methyl­thymidine were found. In general, the mechanism of DNA damage is based on the alkylating methyl­diazonium ion formed from the parent substance.

The majority of micronucleus tests in the bone marrow, intestinal tissue, liver and stomach of rodents yielded positive results. This is probably attributable to clastogenicity. In addition, there is evidence of a mutagenic effect in mammalian somatic cells in vivo. The substance can reach the germ cells and induced methyl DNA adducts in the testes of mice treated subcutaneously.

### 5.7 Carcinogenicity

Numerous studies are available for the carcinogenicity of 1,2-dimethylhydrazine after oral, intraperitoneal, subcutaneous and intramuscular administration. Detailed descriptions are included in an IARC monograph (IARC 1999). A representative selection of these studies is listed below. Inhalation studies are not available.

#### 5.7.1 Short-term studies

In vitro experiments revealed morphological transformation of Syrian golden hamster embryonic cells after exposure to 1,2-dimethylhydrazine concentrations of 0.01 to 10 µg/ml for 8 days (Pienta et al. 1977). A cell transformation assay using human foreskin fibroblasts yielded a positive result (Kumari et al. 1985).

##### 5.7.1.1 Oral administration

In a study, 12 male 21-day-old SD rats were given a single gavage dose of 1,2-dimethylhydrazine of 30 mg/kg body weight. After 36 weeks, half of the animals were found to have adenocarcinomas of the colon (DeRubertis and Craven 1980).

Female BALB/c mice were given a weekly gavage dose of 30 mg 1,2-dimethylhydrazine/kg body weight (unclear ­whether hydrochloride or base is meant; equivalent to 4.3 mg 1,2-dimethylhydrazine/kg body weight and day) for 24 weeks. Thereafter, the animals were observed for 6 (n = 15) or 16 weeks (n = 9). The control group consisted of 15 female mice. At the end of 30 and 40 weeks, 20% and 67% of the animals had blood vessel tumours, 67% and 100% had colon tumours, 27% and 44% had lung tumours, and 13% and 67% had squamous cell carcinomas of the perianal glands, respectively (Izumi et al. 1979).

Male and female rats (21 and 19 animals of the DA strain, 14 and 26 animals of the HS strain, respectively) aged 35 to 45 days were given 10 weekly gavage doses of 30 mg 1,2-dimethylhydrazine dihydrochloride/kg body weight (equivalent to 1.94 mg 1,2-dimethylhydrazine/kg body weight and day). Male and female AS2 rats (16 and 4 animals, respectively) received 10 weekly doses of 10 mg 1,2-dimethylhydrazine dihydrochloride/kg body weight (equivalent to 0.65 mg 1,2-dimethylhydrazine/kg body weight and day). Three treated animals per strain and one animal per strain from the control groups were killed and examined after 4, 9, 15 or 25 weeks, and the remaining after 30 weeks. Two animals of the DA and HS strains died during the experiment. After 30 weeks, the tumour incidences in the colon of male and female DA rats were 10/10 and 13/13 with an average number of tumours per animal of 6.8 and 2.8, respectively. In addition, pineal gland tumours were found in 7/21 male DA rats. The incidences of colon tumours in male and female HS rats were 7/9 and 5/14, respectively. All tumours were classified as adenocarcinomas and developed also in the small intestine with mostly lower incidences. For AS2 rats, the incidences of colon tumours in males and females were 8/10 and 2/4, respectively. One hepatocellular carcinoma, 7 cholangiomas, 3 hepatic angiosarcomas and 2 tumours (not further characterized) of the pineal gland were detected in the twelve AS2 rats (Teague et al. 1981).

In another oral study, the relationship between 1,2-dimethylhydrazine-induced colon tumours and gut-associated lymph­oid tissue was examined. For this purpose, ten 7-week-old male SD rats were given weekly gavage doses of 0 or 15 mg 1,2-dimethylhydrazine dihydrochloride/kg body weight (0.97 mg 1,2-dimethylhydrazine/kg body weight and day) for 5 weeks. Their diet contained either 5% mixed fats or 24% corn oil. The animals were examined 1 week after the end of treatment. No intestinal tumours were found. An additional eight groups of 10 rats each were given weekly gavage doses of 0 or 65 mg/kg body weight of 1,2-dimethylhydrazine dihydrochloride (4.2 mg 1,2-dimethyl­hydrazine/kg body weight and day) for a period of 5 weeks. The groups were additionally fed 5% mixed fat, 24% beef tallow, 24% corn oil or 25% Crisco (vegetable fat) with the diet. The 33 surviving animals were killed and examined 4 months after the first dose. There were no differences in the incidences of intestinal tumours between the groups. A total of 49 adeno­carcinomas developed in the 33 rats. Of these, 71% were polypoid and 29% were sessile. Half of the sessile tumours were associated with intestinal lymphoid tissue. In another experiment, 4 groups of 40 rats received 15 mg/kg body weight 1,2-dimethylhydrazine dihydrochloride (0.97 mg 1,2-dimethylhydrazine/kg body weight and day) administered by gavage once a week for 5 weeks. Groups of 20 animals served as controls. The dose was administered 4 weeks after the start of feeding with different diet compositions (see above). The experiment was terminated 60 weeks after the first administration. A total of 165 colon tumours developed in 159 rats, 38% of which were polypoid and 62% sessile (Nauss et al. 1984).

Thirty-two A/J mice per dose (6–8 weeks old; incidences were not considered separately for the sexes but combined; almost equal numbers of males and females were used for each dose group) were given single gavage doses of 5, 12.5 or 25 mg 1,2-dimethylhydrazine/kg body weight in aqueous solution. The animals were killed and examined 24 weeks after the start of the experiment. 175 animals were used as untreated controls and 100 as vehicle controls. In the dose groups given 5, 12.5 and 25 mg 1,2-dimethylhydrazine/kg body weight, 32, 32 and 16 animals survived and 50%, 56% and 44% developed lung tumours, compared with 23% and 30% in the 2 control groups (Stoner et al. 1984).

##### 5.7.1.2 Subcutaneous injection

Ten male Wistar rats were given weekly subcutaneous injections of 65 mg 1,2-dimethylhydrazine/kg body weight (9.3 mg 1,2-dimethylhydrazine/kg body weight and day) in saline solution for 5 weeks. Five control animals received saline solution. After 10 weeks, the animals were examined. The treated animals gained less weight, and dysplasia and carcinomas were observed in the colon (Jucá et al. 2014).

BALB/c mice (38 males and 47 females) were given weekly subcutaneous injections of 30 mg 1,2-dimethylhydrazine/kg body weight (unclear whether hydrochloride or base was meant; 4.3 mg 1,2-dimethylhydrazine/kg body weight and day) for a period of 16 to 23 weeks and were examined no later than 6 weeks after the end of treatment. Twenty-four male and 23 female mice served as controls. Blood vessel tumours (haemangiomas and haemangioendotheliomas), colon tumours (adenomas and adenocarcinomas), lung tumours (not further specified) and squamous cell carcinomas of the perianal glands developed in 18%, 100%, 5% and 13% of the treated males and in 4%, 100%, 9% and 13% of the treated females, respectively (Izumi et al. 1979).

##### 5.7.1.3 Intraperitoneal injection

In a short-term test, which is no longer common today, 31, 32 and 33 A/J mice (6–8 weeks old) were given single intraperitoneal doses of 5, 12.5 and 25 mg 1,2-dimethylhydrazine/kg body weight in aqueous solution, respectively. Almost equal numbers of male and female animals were used for each dose group; incidences were not considered separately for the sexes but combined. The animals were killed and examined after 24 weeks. 175 animals were used as controls without any treatment and 50 as vehicle controls. None of the treated animals died. Lung adenomas were found in 27%, 25% and 42% of the treated animals, respectively. In the untreated control group, lung tumours developed in 30% of the animals, and in the vehicle control group in 26% (Stoner et al. 1984).

#### 5.7.2 Long-term studies

##### 5.7.2.1 Oral administration

The data from selected studies of the carcinogenicity of 1,2-dimethylhydrazine after oral administration are shown in Table 4.

###### Single exposures

Twenty-eight 7-week-old male F344 rats were given a gavage dose of 35 mg 1,2-dimethylhydrazine dihydrochloride/kg body weight (equivalent to 15.8 mg 1,2-dimethylhydrazine/kg body weight). The animals were observed for 1.5 years. Fourteen animals served as controls. Tumours were found in 78.6% of the exposed animals and none in the control animals. Only 2 tumours were not located in the colon, one of which was in the pineal gland and one in the small intestine. All intestinal tumours were epithelial and located at a maximum distance of 20 cm from the caecum (Schiller et al. 1980).

Four-week-old male F344 rats received a single gavage dose of 35 mg 1,2-dimethylhydrazine dihydrochloride/kg body weight (equivalent to 15.8 mg 1,2-dimethylhydrazine/kg body weight). The groups of treated rats consisted of 22 and 21 animals, and the control groups of 20 animals each. Animals were subsequently fed diets of different composition (cis and trans fats) for 15 months. Seven colon tumours (adenocarcinomas and signet ring cell carcinomas) were found in each of the treated groups, and none in the control animals (Watanabe et al. 1985).

###### Repeated exposure

Female CBA mice (34 animals) were given weekly gavage doses of 8 mg 1,2-dimethylhydrazine/kg body weight for 25 weeks (1.14 mg 1,2-dimethylhydrazine/kg body weight and day). Colon tumours occurred in 23 animals, tumours of the anal region in 14, haemangioendotheliomas of the liver in 7, sarcomas of the uterus in 16, lung tumours in 2. Two groups of female and male (C57BL × CBA)F1 mice (19 animals each) received the substance continuously in drinking water at the same dose for a period of 25 or 20 weeks. Renal adenomas developed in 9 female and 2 male mice, haemangio­endotheliomas in 11 and 7 mice, and blood vessel tumours (renal capsule) in 4 and 16 mice. Only 2 and 3 females had uterine tumours and ovarian tumours (blood vessels), respectively. In the control groups of 19 male and 20 female animals, 4 male mice had lung tumours (not further specified) (IARC 1999).

Seven-week-old Swiss mice (50 males and 50 females) were given 0.001% 1,2-dimethylhydrazine dihydrochloride (10 mg/l) in drinking water for life. This corresponds to about 0.9 mg 1,2-dimethylhydrazine dihydrochloride/kg body weight and day and 0.41 mg 1,2-dimethylhydrazine/kg body weight and day (conversion factor for chronic exposure 0.09 according to EFSA (2012)). The control group consisted of 110 male and 110 female mice. The mortality of the treated animals was increased in a statistically significant manner compared with that in the control group. Haemangiosarcomas were found in 46 male and 49 female treated animals. The average latency periods were 42 and 45 weeks, respectively. Lung adenomas occurred in 12 male and 22 female exposed animals with latency periods of 44 and 49 weeks, respectively (Toth and Wilson 1971).

BALB/c mice (34 males and 37 females) were given 0.001% 1,2-dimethylhydrazine (unclear whether hydrochloride or base was meant) in drinking water (10 mg/l) for life. This corresponds to about 0.9 mg 1,2-dimethylhydrazine/kg body weight and day (conversion factor for chronic exposure 0.09 according to EFSA (2012)). The untreated control animals consisted of 30** **female and 28 male mice. Haemangiosarcomas occurred in 94% of the treated male mice and 97% of the female mice, and lung tumours (not further specified) occurred in 26% of the males and 31% of the females. An additional 29 female BALB/c mice were given 0.004% 1,2-dimethylhydrazine (unclear whether hydrochloride or base was meant) in drinking water (40 mg/l) for 24 weeks (about 6 mg 1,2-dimethylhydrazine/kg body weight and day; conversion factor for subchronic exposure 0.15 according to EFSA (2012)). Haemangiosarcomas were found in 76% of the animals, lung tumours (not further specified) in 14%, and squamous cell carcinomas of the perianal glands in 3%. Ten female BALB/c mice were given the substance in drinking water for 10 weeks (0.008% = 80 mg/l; about 12 mg 1,2-dimethylhydrazine/kg body weight and day; conversion factor for subchronic exposure 0.15 according to EFSA (2012)). Blood vessel tumours were found in 60% of the animals, lung tumours (not further specified) in 50%, squamous cell carcinomas of the perianal glands in 20%, and colon carcinomas in 20% (Izumi et al. 1979).

Eight groups of 50 six-week-old male and 50 six-week-old female mice (Swiss) were given 1,2-dimethylhydrazine dihydro­chloride in drinking water in concentrations in the range from 0.000015625% to 0.002% for life. This corres­ponds to about 0.0064 to 0.82 mg 1,2-dimethylhydrazine/kg body weight and day (conversion factor for chronic exposure 0.09 according to EFSA (2012)). Control animals were not included; instead, reference was made to the control animals of Toth and Wilson (1971). Mortality was increased in a dose-dependent manner. There was a statistically significant and dose-dependent increase in blood vessel tumours. A decrease in the incidence at the highest dose was attributed to the high toxicity in this group. The latency period for tumour development was decreased in a dose-­dependent manner and predominantly angiosarcomas occurred with increasing dose. Furthermore, a statistically significant but not dose-dependent increase in lung tumours was observed in the exposed animals. Colon tumours did not occur (Toth and Patil 1982).

**Tab.4:** Studies of the carcinogenicity of 1,2-dimethylhydrazine (oral administration)

Author:	Schiller et al. 1980								
Substance:	1,2-dimethylhydrazine dihydrochloride								
Species:	**rat**, F344, 28 ♂, 14 ♂ as controls								
Administration route:	single, gavage								
Dose:	15.8 mg 1,2-dimethylhydrazine/kg body weight								
Duration:	1.5 years								
Toxicity:	mortality ↑								
	**Dose (mg/kg body weight)**								
	**0**					**15.8**			
Survivors	14					28			
**Tumours**									
Colon tumours	0/14					21/28 (75%)			
Pineal gland tumours	0/14					1/28 (4%)			
Tumours of the small intestine	0/14					1/28 (4%)			
Author:	Toth and Wilson 1971								
Substance:	1,2-dimethylhydrazine dihydrochloride								
Species:	**mouse**, Swiss Albino, groups of 50 ♂, 50 ♀; 110 ♂, 110 ♀ as controls								
Administration route:	in the drinking water								
Concentration:	0.001% (about 0,41 mg 1,2-dimethylhydrazine/kg body weight and day)								
Duration:	lifespan, maximum 130 weeks								
Toxicity:	mortality ↑								
		**Dose (mg/kg body weight und day)**							
		**0**				**0.41**			
Survivors after 60 weeks	♂	89				0			
	♀	55				0			
**Tumours**									
Haemangiosarcomas	♂	2/110 (1%)				46/50 (92%)			
	♀	4/110 (4%)				49/50 (98%)			
Lung tumours	♂	14/110 (12%)				12/50 (24%)			
	♀	11/110 (10%)				22/50 (44%)			
Author:	Toth and Patil 1982								
Substance:	1,2-dimethylhydrazine dihydrochloride								
Species:	**mouse**, Swiss, 50 ♂, 50 ♀, no controls included								
Administration route:	in the drinking water								
Concentration:	0.000015625%, 0.00003125%, 0,0000625%, 0.000125%, 0.00025%, 0.0005%, 0.001%, 0.002% (about 0.0064, 0.013, 0.026, 0.051, 0.10, 0.21, 0.41, 0.82 mg 1,2-dimethylhydrazine/kg body weight and day)								
Duration:	lifetime, maximum 130 weeks								
Toxicity:	mortality ↑								
		**Dose (mg/kg body weight and day)**							
		**0.0064**	**0.013**	**0.026**	**0.051**	**0.10**	**0.21**	**0.41**	**0.82**
Survivors after 90 weeks	♂	7	16	4	3	1	0	0	0
	♀	24	19	14	12	2	0	0	0
**Tumours** (%)									
Blood vessel tumours (angiomas and sarcomas)	♂	0	6	12	2	58	88	92	78
	♀	2	6	6	24	64	94	98	80
Lung tumours	♂	24	22	20	24	12	24	24	30
	♀	40	26	8	34	20	30	44	22

##### 5.7.2.2 Subcutaneous, intramuscular and intraperitoneal injection

Six male and 3 female monkeys (Macaca fascicularis) were given subcutaneous injections of 16 mg 1,2-dimethyl­hydrazine/kg body weight 3 times a month for 2 years. Experiments were terminated 275 weeks after the first injection. Two male monkeys developed adenocarcinomas in the colon 34 and 47 weeks after the start of the experiment and 1 female monkey developed a fibromyoma in the uterus after 55 weeks (Beniashvili et al. 1992).

Male Syrian golden hamsters (n = 40) were given weekly intramuscular injections of 19.5 mg 1,2-dimethylhydrazine/kg body weight for 3 weeks (2.8 mg 1,2-dimethylhydrazine/kg body weight and day). Twelve hamsters served as the untreated control group. All animals were observed until their natural death. Fifteen treated animals died before the end of the experiment. Among the 25 surviving hamsters, 9 (36%) developed gastrointestinal tumours: 1 adenocarcinoma of the stomach, 1 adenocarcinoma of the duodenum, 4 adenocarcinomas of the ileum, and 3 adenocarcinomas of the colon. Five animals had hepatocellular carcinomas (IARC 1999).

A group of 60 female CF1 mice were given weekly subcutaneous injections of 9.1 mg 1,2-dimethylhydrazine/kg body weight for 6 weeks. This corresponds to a dose of 1.3 mg 1,2-dimethylhydrazine/kg body weight and day. After 45 weeks, the surviving 43 animals were killed and examined. In 36 of the 43 mice (83%), an average of 2.3 colonic tumours per animal had formed. The 3-cm segment of the colon above the anus (distal region) contained 61% of all neoplasms. Other tumours, not recorded numerically, occurred in 13 of the 43 animals. The tumours showed the full spectrum of neoplastic lesions from morphologically benign polyps to adenocarcinomas (IARC 1999).

Female 10 to 12-week-old mice of 8 strains were given weekly subcutaneous injections of 8 mg 1,2-dimethylhydrazine/kg body weight (1.14 mg 1,2-dimethylhydrazine/kg body weight and day) for 25 weeks and were observed for a further 25 weeks. Adenomas and carcinomas of the colon were induced in all strains, with the highest incidence in BALB/c mice (93.3%) and the lowest incidence in C3HA mice (30.9%). Tumours (not further specified) of the anus region and clitoral glands occurred in all animal strains (incidences in the range from 24% to 63%). Uterine sarcomas developed in 37.5%, 40.7%, 20.7%, 7.7% and 2.7% of C3H, CBA, (C57BL × CBA)F1, AKR and C57BL/6j mice, respectively. None of the above tumours was found in untreated mice of the same strains (Turusov et al. 1982).

Two groups of 39 and 27 female CBA mice, respectively, were given weekly 1,2-dimethylhydrazine doses of 8 mg/kg body weight for 25 weeks (1.14 mg 1,2-dimethylhydrazine/kg body weight and day); the first group was treated intraperitoneally the second subcutaneously. Carcinomas of the colon occurred in 24 and 19 animals, tumours of the anal region in 22 and 16, haemangioendotheliomas of the liver in 13 and 5, and tumours of the uterus in 11 in both groups. Liver adenomas were diagnosed in only 2 animals in the subcutaneous group. In the group treated intraperitoneally, 3 animals developed tumours in the blood vessels of the renal capsule and 2 developed ovarian tumours. Three other groups of 28, 29 and 28 female CBA mice were given subcutaneous doses of 1,2-dimethylhydrazine either at a dose level of 16 mg/kg body weight (1.14 mg/kg body weight and day) every 2 weeks or 8 mg/kg body weight (1.14 mg/kg body weight and day) weekly or 1.14 mg/kg body weight daily for 30 weeks. Tumours developed in the anal region of 17, 23 and 6 animals, and haemangioendotheliomas in 9, 8 and 5 animals, respectively. In the former 2 groups, colon tumours (not further specified) developed in 17 and 21 animals and uterine sarcomas in 12 and 14 animals, respectively. Blood vessel tumours in the ovaries occurred in 2 animals after weekly administration. Two additional groups of 36 and 69 female CBA mice were given subcutaneous injections of either 8 mg/kg body weight weekly (equivalent to a dose of 1.14 mg 1,2-dimethylhydrazine/kg body weight and day) or 1.14 mg/kg body weight daily, respectively, for 10 weeks. A control group of 40 mice was given saline. In the first 2 groups, colon tumours (not further specified) developed in 12 and 3 animals, tumours in the anal region in 21 and 17 animals, renal adenomas in 8 and 43 animals, liver adenomas in 16 and 28 animals, haemangioendotheliomas in 4 and 29 animals, uterine sarcomas in 6 and 12 animals, and lung tumours in 7 and 15 animals, respectively. In the group treated daily, blood vessel tumours in the ovaries occurred in 4 animals. In the control group, only 1 animal was found to have a liver adenoma. In the same study, groups of 33 female and 48 male (C57BL × CBA)F1 mice were given weekly subcutaneous injections of the substance for 25 and 20 weeks, respectively (dose not specified, probably also 8 mg/kg body weight; equivalent to a dose of 1.14 mg 1,2-dimethylhydrazine/kg body weight and day). Two additional groups of 20 and 19 female and male F1 mice, respectively, served as controls. In the groups with weekly subcutaneous administration, colon tumours (not further specified) developed in 24 and 41 animals, tumours in the anal region (not further specified) in 10 and 14 animals, renal adenomas in 6 and 21 animals, liver adenomas in 3 and 11 animals, haemangioendotheliomas in 16 and 6 animals, lung tumours (not further specified) in 3 animals in both groups, blood vessel tumours (renal capsule) in 1 and 9 animals, respectively. In addition, uterine sarcomas and blood vessel tumours in the ovaries occurred in 19 and 5 animals, respectively. In the continuous dose groups, renal adenomas developed in 9 female and 2 male mice, haemangioendotheliomas in 11 and 7 mice, and blood vessel tumours (renal capsule) in 4 and 16 mice. Only 2 and 3 females had uterine tumours and ovarian tumours (blood vessels), respectively. In the control groups, 4 male mice had lung tumours (not further specified) (IARC 1999).

In another study, CBA mice were given 8, 16 or 32 weekly subcutaneous injections of 8 mg 1,2-dimethylhydrazine/kg body weight (1.14 mg/kg body weight and day) and were observed for their lifetime. Twenty to 29 animals of both sexes were used in the 3 groups. A group without treatment served as controls (44 female, 48 male mice). Vascular and epithelial renal tumours (3.4%–85%), colon tumours (20.7%–55%), tumours of the anal region (25%–65%), liver haem­angioendotheliomas (15%–36%) and uterine sarcomas (31%–60%) occurred. Some tumour incidences increased with the duration of treatment. The incidence of hepatomas was decreased compared with that in the controls (Turusov et al. 1988).

Eight to nine-week-old female SWR mice were given weekly intraperitoneal injections of 6.8 mg 1,2-dimethylhydrazine/kg body weight for 1, 5, 10 or 20 weeks, and the animals were subsequently observed for 2 years. Groups of 36, 21, 24 or 32 animals were treated and 39 control animals were used. The mortality and the incidences of colon adenocarcinomas (0%–87%) in the treated animals increased with the dose. The majority of tumours occurred in the distal colon. Anal cysts were increased dose-dependently in all treated groups (Jackson et al. 1999).

#### 5.7.3 Summary

In numerous carcinogenicity studies, colon tumours developed in rats and mice after oral administration of 1,2-dimethylhydrazine, and in rats even after a single dose. In mice, blood vessel tumours, tumours of the perianal glands, uterine tumours and lung tumours were induced. These tumour types were likewise observed after subcutaneous (rat, mouse) and intraperitoneal (mouse) administration. In monkeys, colon tumours likewise developed after subcutaneous injection. In hamsters, 1,2-dimethylhydrazine induced colon and liver tumours after intramuscular administration.

## 6 Manifesto (MAK value/classification)

The critical effect is the carcinogenic effect resulting from genotoxicity.

**Carcinogenicity. **1,2-Dimethylhydrazine has been shown to be genotoxic in numerous studies in vitro and in vivo. The mechanism is based on DNA methylation by the methyldiazonium ion which is formed during metabolism. In particular, this involves the formation of N7-methylguanine and O6-methylguanine, which leads to base mismatches in the DNA. In rats and mice, mainly colon tumours developed after oral administration, in rats even after a single dose. In mice, blood vessel tumours, and tumours of the perianal glands, the uterus and the lungs were induced. These tumour types were observed also after subcutaneous (rat, mouse) and intraperitoneal (mouse) administration. The substance has a clear carcinogenic potential. The classification of 1,2-dimethylhydrazine in Carcinogen Category 2 has therefore been retained.

**MAK value. **Even low doses of 1,2-dimethylhydrazine (0.32 mg/kg body weight and day) caused liver damage, haemorrhage and fibrosis in dogs. The carcinogenicity is based on genotoxic effects. It is not possible to establish an exposure–risk relationship. A MAK value cannot be derived and the substance is thus not assigned to a peak limitation category.

**Prenatal toxicity. **There are no studies of prenatal developmental toxicity according to valid test guidelines available for 1,2-dimethylhydrazine. Since a MAK value cannot be derived, assignment to a pregnancy risk group is not applicable.

**Germ cell mutagenicity. **Studies with germ cells are not available. Based on in vitro and in vivo studies, a methylating effect of 1,2-dimethylhydrazine on DNA can be assumed. Clastogenicity in somatic cells is to be assumed in the light of all the available data and mutagenicity in vivo has been demonstrated. The accessibility of germ cells has been shown, and indirect evidence of a genotoxic effect was provided by the detection of methylated DNA adducts in the testes of treated animals. Taking into consideration the structurally related substance 1,1-dimethylhydrazine, for which germ cell mutagenicity has been demonstrated and which has been classified in Category 3 A for germ cell mutagens (Hartwig and MAK Commission 2024), 1,2-dimethylhydrazine has likewise been classified in Category 3 A for germ cell mutagens.

**Absorption through the skin. **Valid studies of the absorption of 1,2-dimethylhydrazine through the skin are not available. Due to the low dermal LD_50_ values of less than 1000 mg/kg body weight (Section 5.1.3) and because 1,2-dimethylhydrazine is a genotoxic substance, designation with an “H” (for substances which can be absorbed through the skin in toxicologically relevant amounts) has been retained.

**Sensitization. **1,2-Dimethylhydrazine has been designated with “S” and “Sh” (for substances which cause sensitization of the skin) since 1979. However, there are no data available for sensitizing effects on the skin or the respiratory tract. According to the criteria for the evaluation of sensitizing substances (see Section IV of the List of MAK and BAT Values (DFG 2021)), a close structural relationship with similar substances that have been classified as sensitizing substances is not sufficient in itself to assume that the substance is likely to have a sensitizing effect if additional positive findings are not available. However, in the case of the methylhydrazines, it seems plausible that they would cause contact sensitization because of the close structural similarity to hydrazine, which is known to be a pronounced contact allergen and has been designated with “Sh”. 1,2-Dimethylhydrazine was assigned the designation “Sh” in 1979 as a precautionary measure. This designation has been retained because there are no data available that demonstrate that 1,2-dimethylhydrazine is not a contact allergen.
